# BMX controls 3βHSD1 and sex steroid biosynthesis in cancer

**DOI:** 10.1172/JCI163498

**Published:** 2023-01-17

**Authors:** Xiuxiu Li, Michael Berk, Christopher Goins, Mohammad Alyamani, Yoon-Mi Chung, Chenyao Wang, Monaben Patel, Nityam Rathi, Ziqi Zhu, Belinda Willard, Shaun Stauffer, Eric Klein, Nima Sharifi

**Affiliations:** 1 Genitourinary Malignancies Research Center, Lerner Research Institute,; 2 Center for Therapeutics Discovery, Lerner Research Institute,; 3 Department of Inflammation and Immunity, Lerner Research Institute,; 4 Mass Spectrometry Core, Lerner Research Institute,; 5 Department of Urology, Glickman Urological and Kidney Institute, and; 6 Department of Hematology and Oncology, Taussig Cancer Institute, Cleveland Clinic, Cleveland, Ohio, USA.

**Keywords:** Oncology, Prostate cancer

## Abstract

Prostate cancer is highly dependent on androgens and the androgen receptor (AR). Hormonal therapies inhibit gonadal testosterone production, block extragonadal androgen biosynthesis, or directly antagonize AR. Resistance to medical castration occurs as castration-resistant prostate cancer (CRPC) and is driven by reactivation of the androgen-AR axis. 3β-hydroxysteroid dehydrogenase-1 (3βHSD1) serves as the rate-limiting step for potent androgen synthesis from extragonadal precursors, thereby stimulating CRPC. Genetic evidence in men demonstrates the role of 3βHSD1 in driving CRPC. In postmenopausal women, 3βHSD1 is required for synthesis of aromatase substrates and plays an essential role in breast cancer. Therefore, 3βHSD1 lies at a critical junction for the synthesis of androgens and estrogens, and this metabolic flux is regulated through germline-inherited mechanisms. We show that phosphorylation of tyrosine 344 (Y344) occurs and is required for 3βHSD1 cellular activity and generation of Δ^4^, 3-keto-substrates of 5α-reductase and aromatase, including in patient tissues. BMX directly interacts with 3βHSD1 and is necessary for enzyme phosphorylation and androgen biosynthesis. In vivo blockade of 3βHSD1 Y344 phosphorylation inhibits CRPC. These findings identify what we believe to be new hormonal therapy pharmacologic vulnerabilities for sex-steroid dependent cancers.

## Graphical abstract

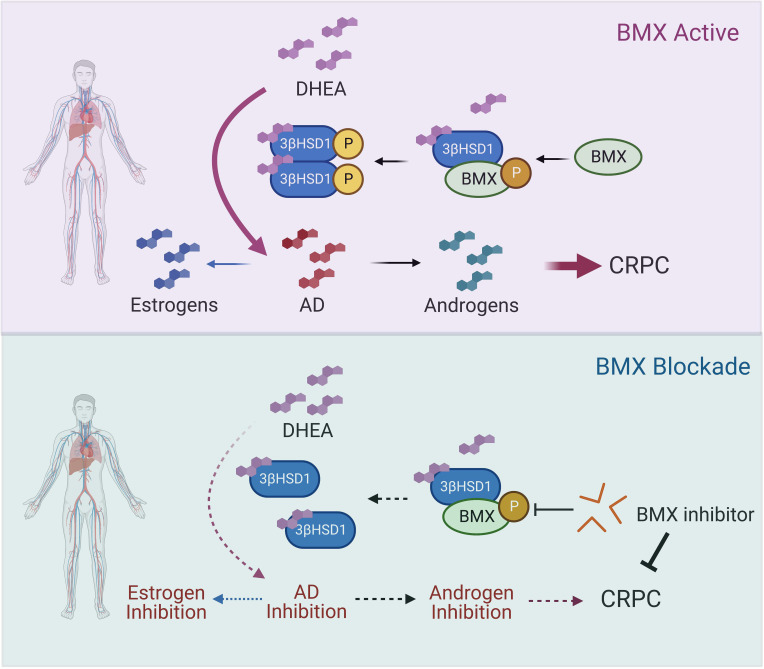

## Introduction

Metastatic prostate cancer requires systemic therapy. Androgen deprivation therapy (ADT) with medical or surgical castration is the front-line treatment and is now frequently combined with other therapies; however, it eventually fails, and disease almost always progresses as castration-resistant prostate cancer (CRPC). Nearly all prostate cancer deaths are due to CRPC (1, 2). Intratumoral androgen biosynthesis is required to drive CRPC progression (3–6). The enzyme 3β-hydroxysteroid dehydrogenase-1 (3βHSD1), which catalyzes the initial, irreversible, and rate-limiting step in the conversion of the adrenal-derived steroid dehydroepiandrosterone (DHEA) to dihydrotestosterone (DHT) — the most potent natural stimulus of the AR — is a critical enzymatic gatekeeper that confers tumors with the ability to harness adrenal androgens (7). 3βHSD1 catalyzes 2 reactions on the steroid backbone — 3β-OH to 3-keto oxidation and Δ^5^ to Δ^4^ isomerization — that together make this metabolic step effectively irreversible (8–10). *HSD3B1*, which encodes for 3βHSD1, has a common missense-encoding germline variation that promotes CRPC (11, 12). In men with prostate cancer, the adrenal-restrictive *HSD3B1*(1245A) allele limits conversion from DHEA to potent androgens, whereas the adrenal-permissive *HSD3B1*(1245C) allele harbors a 367T missense protein that stabilizes the enzyme, enables potent androgen synthesis, and leads to more rapid development of resistance to ADT and next-generation hormonal therapies, thus shortening overall survival (13–17). Consequently, 3βHSD1 drives resistance to several lines of hormonal therapy, and thus, the identification of 3βHSD1 as a clinical resistance driver has made it a genetically validated target in men with prostate cancer. No phosphorylation sites are known to be required for 3βHSD1 activation. Furthermore, there are no known clinically feasible methods to effectively block 3βHSD1.

Epithelial and endothelial tyrosine kinase (Etk, also known as BMX) is a nonreceptor tyrosine kinase that has been implicated in various biological processes, including proliferation, differentiation, apoptosis, and cell migration. BMX expression is upregulated in response to androgen ablation in prostate cancer, suggesting that Etk/BMX may be involved in the development and progression of CRPC (18–20). However, the downstream mechanisms of BMX action remain elusive, and it has no known role in regulating androgen synthesis.

In this study, we identified multiple phosphorylation sites on 3βHSD1 and found that phosphorylation of a single tyrosine (Y) is required for 3βHSD1 activation, and BMX is the necessary kinase. BMX blockade inhibits metabolism of (Δ^5^, 3β-OH) DHEA to (Δ^4^, 3-keto) Δ^4^-androstenedione (Δ^4^-AD), which is the major substrate for steroid-5α-reductase (SRD5A) and aromatase, downstream enzymes required for potent androgen and estrogen synthesis, respectively. BMX therefore regulates the generation of the Δ^4^, 3-keto-steroid structure, which is necessary for all potent sex steroids. Further, we show that targeting BMX is a potential treatment strategy for CRPC.

## Results

### Phosphorylation of 3βHSD1 Y344 is required for DHEA metabolism.

**Figure 1:**
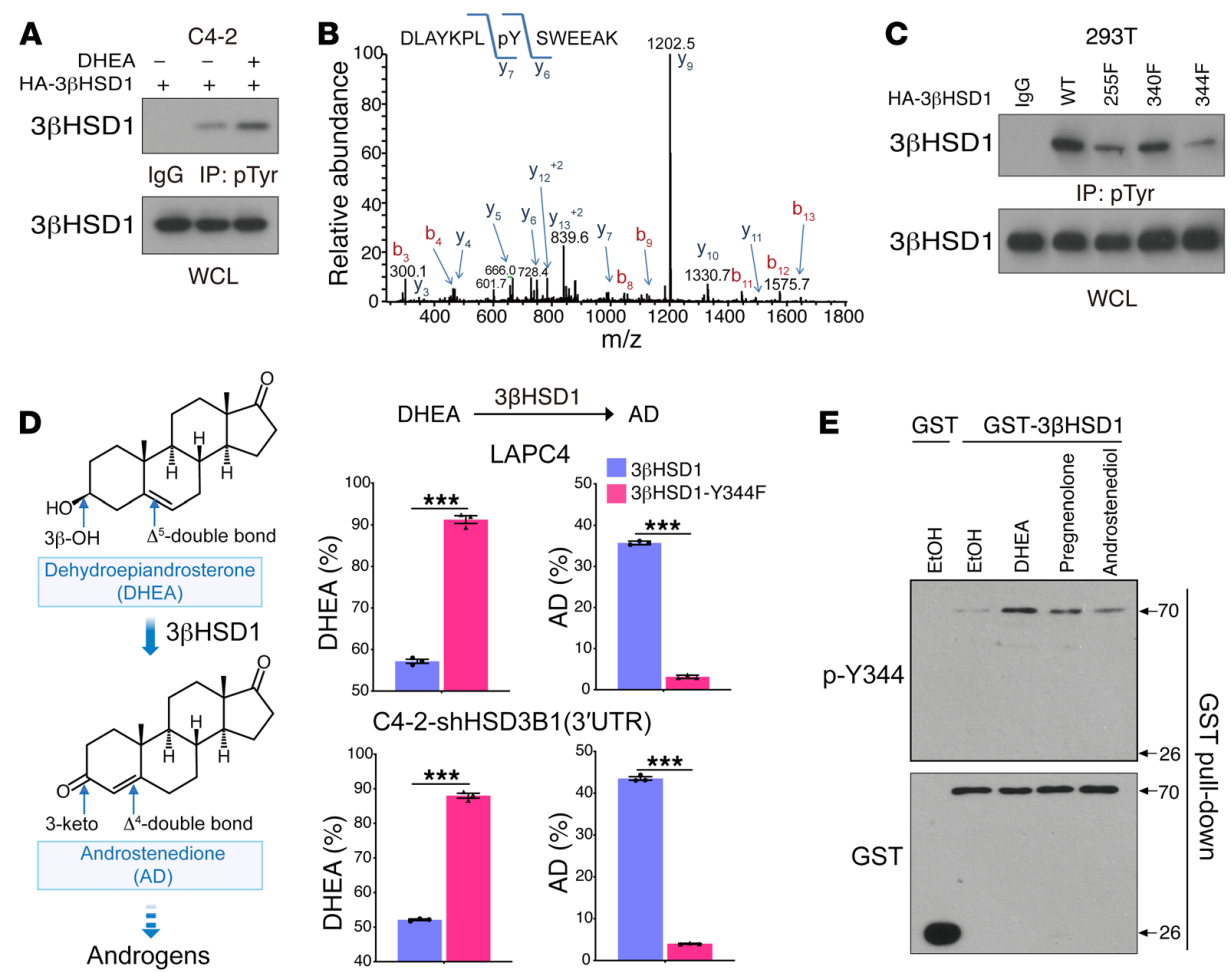
3β-hydroxysteroid dehydrogenase pY344 is required for DHEA metabolism. (**A**) C4-2 cells overexpressing HA-3β-hydroxysteroid dehydrogenase (HA-3βHSD1) were treated with or without DHEA for 1 hour. Pan-phospho-tyrosine (pTyr) was detected by IP and Western blot. Control lysate without DHEA treatment was used for the IgG control. (**B**) 3βHSD1-GST was transfected, and cells were treated with DHEA for 1 hour. GST pull-down complexes were immunoblotted, and the indicated phosphopeptides on human 3βHSD1 were identified by LC-MS/MS. A doubly charged peptide with a mass of 896.91 Da was identified in the survey analysis of GST-HSD3B1. The CID spectra for this peptide are dominated by singly charged C-terminal y ions. The mass difference between y_7_ and y_6_ is consistent with modification at Y344. (**C**) Cells were transfected with HA-3βHSD1 mutants and treated as in (**A**). Lysate from WT-expressing cells was used for the IgG control. (**D**) 3βHSD1 enzyme activity was assessed by analyzing DHEA metabolism by HPLC. Cells were transfected with Flag-3βHSD1 mutants and subsequently treated with [^3^H]-DHEA for 4 hours, followed by steroid extraction from media, steroid separation, and quantitation with HPLC. The experiment was done in triplicate and repeated in independent experiments. Shown are the steroid sites of 3βHSD1 biochemical modification. Mean ± SEM represents 3 replicates in 1 experiment. Three independent experiments were performed. ***P < 0.001 (unpaired 2-tailed *t* test). (**E**) C4-2 cells overexpressing 3βHSD1-GST were treated with ethanol or DHEA, pregnenolone, or androstenediol for 1 hour. GST pull-down complexes were immunoblotted with a phospho-3βHSD1-Y344 antibody.

To test whether phosphorylation is necessary for 3βHSD1 activity, we overexpressed HA-3βHSD1 in C4-2 cells. Immunoblotting analyses of immunoprecipitated HA-3βHSD1 with anti-phospho-tyrosine antibodies showed that 3βHSD1 undergoes tyrosine phosphorylation, which is induced in the presence of its substrates, DHEA, pregnenolone, and androstenediol (Figure 1A and Supplemental Figure 1, A and B; supplemental material available online with this article; https://doi.org/10.1172/JCI163498DS1). Mass spectrometry analyses of immunoprecipitated GST-3βHSD1 expressed in C4-2 cells showed that 3βHSD1 was phosphorylated at Y255 and Y344 (Figure 1, B–D). This result was further supported by immunoblotting analyses. Mutation of Y344 or Y255 to phenylalanine (21) reduced tyrosine phosphorylation of 3βHSD1 (Figure 1C). Further, we found that phosphorylation of Y344 but not Y255 was required for 3βHSD1 cellular activity because DHEA metabolism was significantly reduced after mutation to phenylalanine (Figure 1D and Supplemental Figure 1E). To gain further insight about the mechanisms of this modification, we custom-designed an anti-phospho-3βHSD1 Y344 (anti-3βHSD1 pY344) antibody. After determining that it had a high level of specificity (Supplemental Figure 1F), we used it to detect 3βHSD1 pY344 in C4-2 and LNCaP cells. We found that 3βHSD1 pY344 was induced by DHEA treatment (Figure 1E). Collectively, these results indicated that Y344 phosphorylation was essential for 3βHSD1 enzyme activity in cells.

### BMX was required for DHEA metabolism by 3βHSD1.

**Figure 2:**
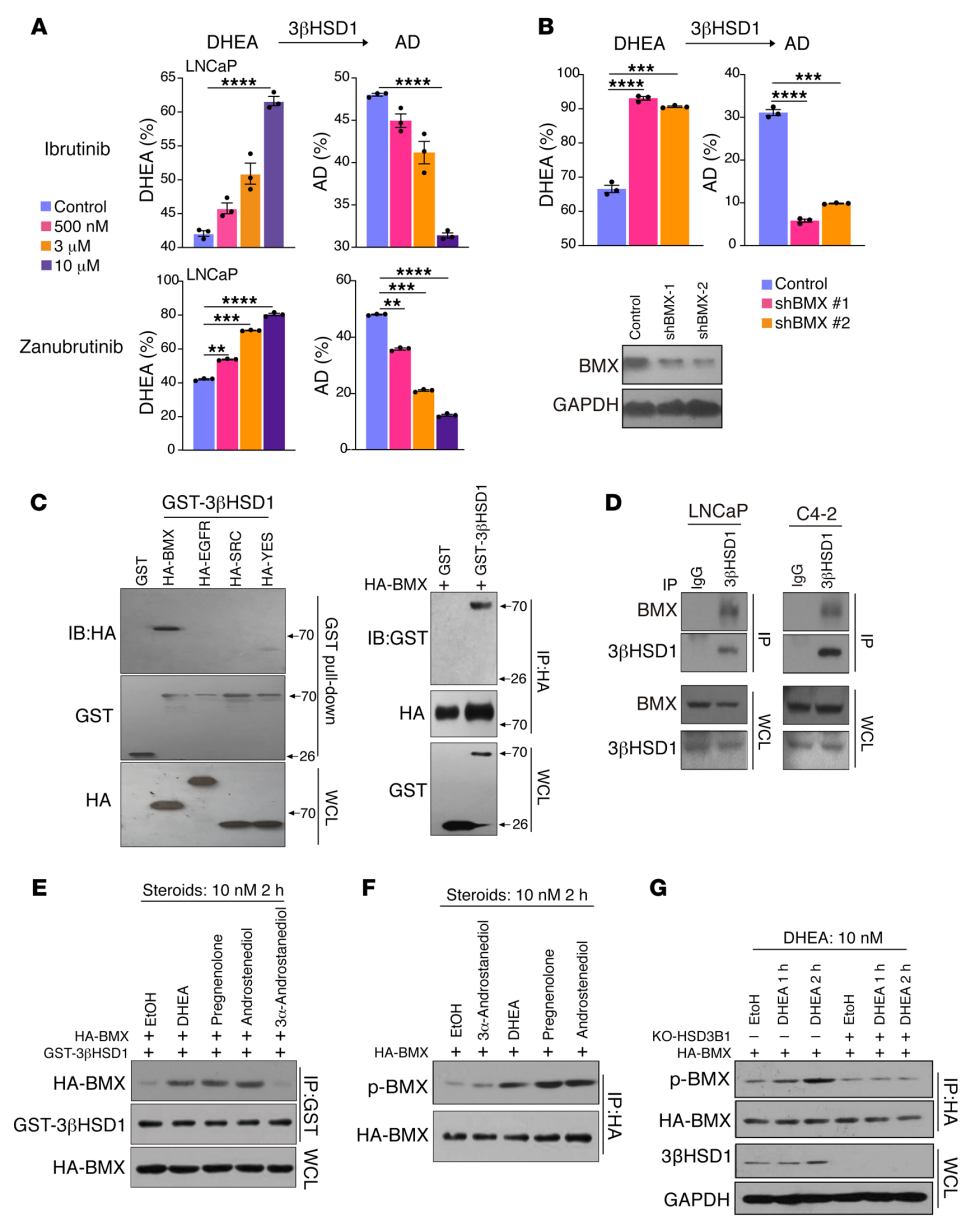
BMX is required for DHEA metabolism by 3βHSD1. (**A**) LNCaP cells were treated with ibrutinib or zanubrutinib for 1 hour and subsequently treated with [^3^H]-DHEA for 5 hours, followed by steroid extraction from media and steroid separation and quantitation with HPLC. The experiment was done in triplicate and repeated in independent experiments. Mean ± SEM represents 3 replicates in 1 experiment. 3 independent experiments were performed. **P < 0.01, ***P < 0.001, ****P < 0.0001 (1-way ANOVA test with Dunnett’s multiple comparisons test). (**B**) Cells stably expressing shNT or 2 shRNA sequences against BMX were treated with [^3^H]-DHEA for 6 hours and analyzed as in (**A**). Mean ± SEM represents combined data from 3 biological independent replicates performed in technical triplicate. ***P < 0.001, ****P < 0.0001 (1-way ANOVA test with Dunnett’s multiple comparisons test). (**C**) 293T cells were transiently cotransfected with HA-BMX, EGFR, SRC, or YES and GST-3βHSD1, followed by GST pull-down and Western blot (left). 293T cells were transiently cotransfected with HA-BMX and GST-3βHSD1, followed by HA immunoprecipitation and Western blot (right). (**D**) LNCaP and C4-2 cells were cultured, protein collected, and immunoprecipitation and Western blot performed for endogenously expressed proteins. WCL blots run in parallel, contemporaneously, using identical samples are shown. (**E**) LNCaP cells were transiently cotransfected with HA-BMX, and GST-3βHSD1cells were starved with medium containing 10% charcoal-stripped FBS for 24 hours, then treated with steroids for 2 hours, followed by GST-pull down and Western blot to detect interaction of HA-BMX and GST-3βHSD1. (**F**) LNCaP cells were starved as in (**E**), then transfected with HA-BMX and treated with steroids for 2 hours; p-BMX was detected by Western blot. Blots run in parallel, contemporaneously, using identical samples are shown. (**G**) Stable C4-2 cell lines with *HSD3B1* gRNA or control gRNA were transfected with HA-BMX, starved as in (**E**) and treated with DHEA for 2 hours; p-BMX was detected by Western blot.

To identify the kinase that phosphorylates 3βHSD1, we used a kinase inhibitor library to screen in an unbiased fashion for pharmacologic inhibitors that block [^3^H]-DHEA metabolism (Supplemental Figure 2A). HPLC analyses showed that pharmacologic inhibitors of BMX or PDGFR block [^3^H]-DHEA metabolism to AD. Kinase prediction based on Y344 using the phosphoNET Kinase Predictor (http://www.phosphonet.ca/kinasepredictor.aspx?uni=P14060&ps=Y344) showed that BMX had the greatest potential to phosphorylate 3βHSD1 at Y344. Further, we tested several BTK/BMX inhibitors, with HPLC analyses showing that zanubrutinib or ibrutinib treatment significantly decreased DHEA metabolism in multiple prostate cancer (LNCaP, C4-2, and VCaP) cell lines (Figure 2A and Supplemental Figure 2, B–D). The 3β-OH → 3-keto and Δ^5^ → Δ^4^ reactions catalyzed by 3βHSD1 to synthesize AD not only serve to make this a substrate of SRD5A (22, 23), these reactions are also essential to generate 3-keto, Δ^4^-steroid substrates (e.g., AD and testosterone) for aromatase and estrogen biosynthesis (24). Thus, we examined the effect of BMX inhibition on estrogen synthesis from DHEA as a precursor. We found that zanubrutinib also inhibits the production of estradiol from DHEA (Supplemental Figure 2E).

We generated LNCaP cells stably expressing shNT (nontargeting control) or shRNA sequences against BMX (shBMX) and found that knocking down BMX also inhibited DHEA metabolism (Figure 2B). We next examined the interaction between 3βHSD1 and BMX. Coimmunoprecipitation assays showed that 3βHSD1 interacted with BMX (Figure 2, C and D), and this interaction was induced by the 3βHSD1 substrates DHEA, pregnenolone, and androstenediol (Figure 2E). Moreover, we found that 3βHSD1 substrates induced the phosphorylation of BMX (Figure 2F) and that 3βHSD1 substrates are needed for the induction of BMX phosphorylation (Figure 2G). In the absence of 3βHSD1, DHEA failed to activate the phosphorylation of BMX, suggesting that DHEA-mediated 3βHSD1-BMX interaction is needed for BMX activation. Taken together, our findings indicated that BMX was required for DHEA metabolism by 3βHSD1.

### BMX directly phosphorylated 3βHSD1 at Y344.

**Figure 3:**
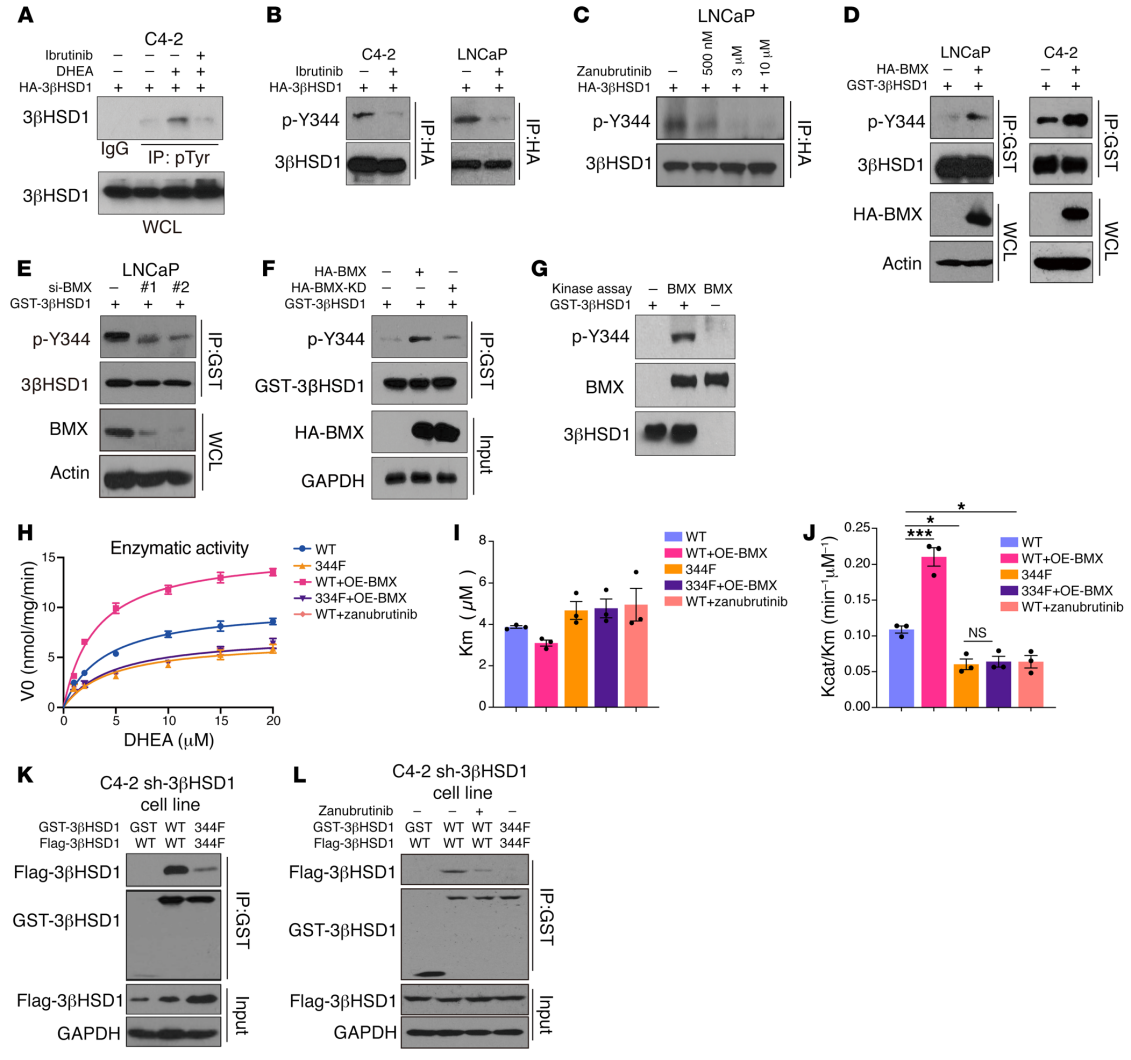
BMX directly binds 3βHSD1 and phosphorylates Y344. (**A**) LNCaP cells overexpressing 3βHSD1 were treated with ibrutinib for 1.5 hours with or without DHEA for 0.5 hours. Pan-phospho-tyrosine (pTyr) was detected by immunoprecipitation and Western blot. (**B** and **C**) Cells overexpressing 3βHSD1 were treated with ibrutinib or zanubrutinib for 3 hours, and DHEA for 1 hour (**B**) or 2 hours (**C**). Phospho-3βHSD1-Y344 was detected by immunoprecipitation and Western blot. Blots run in parallel, contemporaneously, using identical samples are shown. (**D**) Cells with cooverexpression of GST-3βHSD1 and HA-BMX or vehicle were treated with DHEA for 1 hour. Phospho-3βHSD1-Y344 was detected by immunoprecipitation and Western blot. Actin blots, serving as loading controls, were run in parallel, contemporaneously using identical samples with other blots. (**E**) Cells overexpressing GST-3βHSD were transfected with siNT or 1 of 2 siRNA sequences against BMX; phospho-3βHSD1-Y344 was detected by GST pull-down and Western blot. 3βHSD1 blots, serving as loading controls, were run in parallel, contemporaneously using identical samples with other blots. (**F**) Cells with cooverexpression of GST-3βHSD and HA-BMX or HA-BMX-KD (kinase dead) were treated with DHEA for 1 hour. Phospho-3βHSD1-Y344 was detected by immunoprecipitation and Western blot. (**G**) GST-3βHSD or HA-BMX was purified from 293T cells; 3βHSD1-GST was dephosphorylated using phosphatase in vitro, followed by a kinase assay and Western blot. (**H**–**J**) 293T cells were transfected with 3βHSD1 or Y344F mutant with or without cooverexpressed HA-BMX. 3βHSD1 or 3βHSD1-Y344F mutant was immunopurified, and an NAD+ turnover assay was performed. Mean ± SEM represents combined data from 3 independent experiments. *P < 0.05, ***P < 0.001 (1-way ANOVA with Bonferroni’s multiple comparisons test). (**K**) GST-tagged and flag-tagged WT or Y344F-3βHSD1 were transfected into C4-2 cells, GST pull-down was performed, and flag-tagged 3βHSD1 was detected by Western blot. Cells were treated with DHEA for 2 hours. (**L**) GST-tagged and flag-tagged WT or Y344F-3βHSD1 were transfected into C4-2 cells, GST pull-down was performed, and flag-tagged 3βHSD1 was detected by Western blot. Cells were treated with DHEA for 2 hours; zanubrutinib (10 μM) treatment was 24 hours.

To investigate the role of BMX in phosphorylation of 3βHSD1, we inhibited BMX activity in C4-2 and LNCaP cells. Immunoblotting analyses of immunoprecipitated HA-3βHSD1 with anti-phospho-tyrosine and anti-3βHSD1 pY344 antibodies showed that the BMX inhibitors ibrutinib and zanubrutinib blocked DHEA-induced phosphorylation of 3βHSD1 (Figure 3, A–C). We overexpressed HA-BMX in C4-2 and LNCaP cells. Immunoblotting analyses of immunoprecipitated HA-3βHSD1 with anti-3βHSD1 pY344 antibodies showed that BMX enhanced phosphorylation of 3βHSD1 pY344 (Figure 3D). Moreover, knockdown of BMX substantially reduced 3βHSD1 Y344 phosphorylation with DHEA stimulation in LNCaP cells (Figure 3E). Furthermore, overexpression of a kinase-dead BMX mutant (KD-BMX) in C4-2 cells strikingly failed to stimulate 3βHSD1 Y344 phosphorylation that was induced by WT-BMX (Figure 3F). To determine whether BMX directly phosphorylated 3βHSD1, we carried out in vitro kinase assays by mixing purified GST-3βHSD1 and HA-BMX. The assays showed that BMX directly phosphorylated 3βHSD1 at Y344 (Figure 3G). These results indicated that BMX was the kinase that directly phosphorylated 3βHSD1 at Y344.

### 3βHSD1 pY344 enhanced dimerization.

We next investigated how Y344 phosphorylation promotes 3βHSD1 cellular enzymatic activity. We purified 3βHSD1 or 3βHSD1-Y344F mutant and performed an NAD+ turnover assay in vitro. The results showed that WT enzyme purified from zanubrutinib-treated cells and the phospho-mutant of 3βHSD1 had lower catalytic activity (Kcat/Michaelis constant [Kcat/Km]); WT enzyme purified from cells with overexpressed BMX had a marginal increase in catalytic activity (Kcat/Km); and no significant increase in Kcat/Km was observed for 3βHSD1 phospho-mutant expressed in cells with overexpressed BMX (Figure 3, H, I and J). 3βHSD1 phosphorylation did not significantly affect the Km of DHEA. These results suggest that in a purified in vitro context, phosphorylation appears to have minimal influence on observed enzymatic activity. Furthermore, phosphorylation of Y344 at 3βHSD1 had no effect on its protein expression or degradation (Supplemental Figure 3, A and C), nor did zanubrutinib affect the level of 3βHSD1 protein expression (Supplemental Figure 3B). Some hydroxysteroid dehydrogenase enzymes are known to exist as dimers (25). To further investigate how Y344 phosphorylation may influence 3βHSD1 activity, we tested whether Y344 phosphorylation regulates 3βHSD1-dimer formation in C4-2 cells cotransfected with Flag-3βHSD1 and GST-3βHSD1 and treated with DHEA. Coimmunoprecipitation of Flag-3βHSD1 and GST-3βHSD1 was inhibited by blocking Y-phosphorylation using the Y344F mutation, as well as with zanubrutinib treatment, which suggested that 3βHSD1 pY344 enhanced dimerization in cells (Figure 3, K and L). These results suggested that phosphorylation may promote cellular dimerization and thus enhance enzymatic activity of 3βHSD1 in a cellular context.

### BMX blockade and inhibition of 3βHSD1 phosphorylation impeded expression of androgen-regulated genes and prostate cancer proliferation.

**Figure 4:**
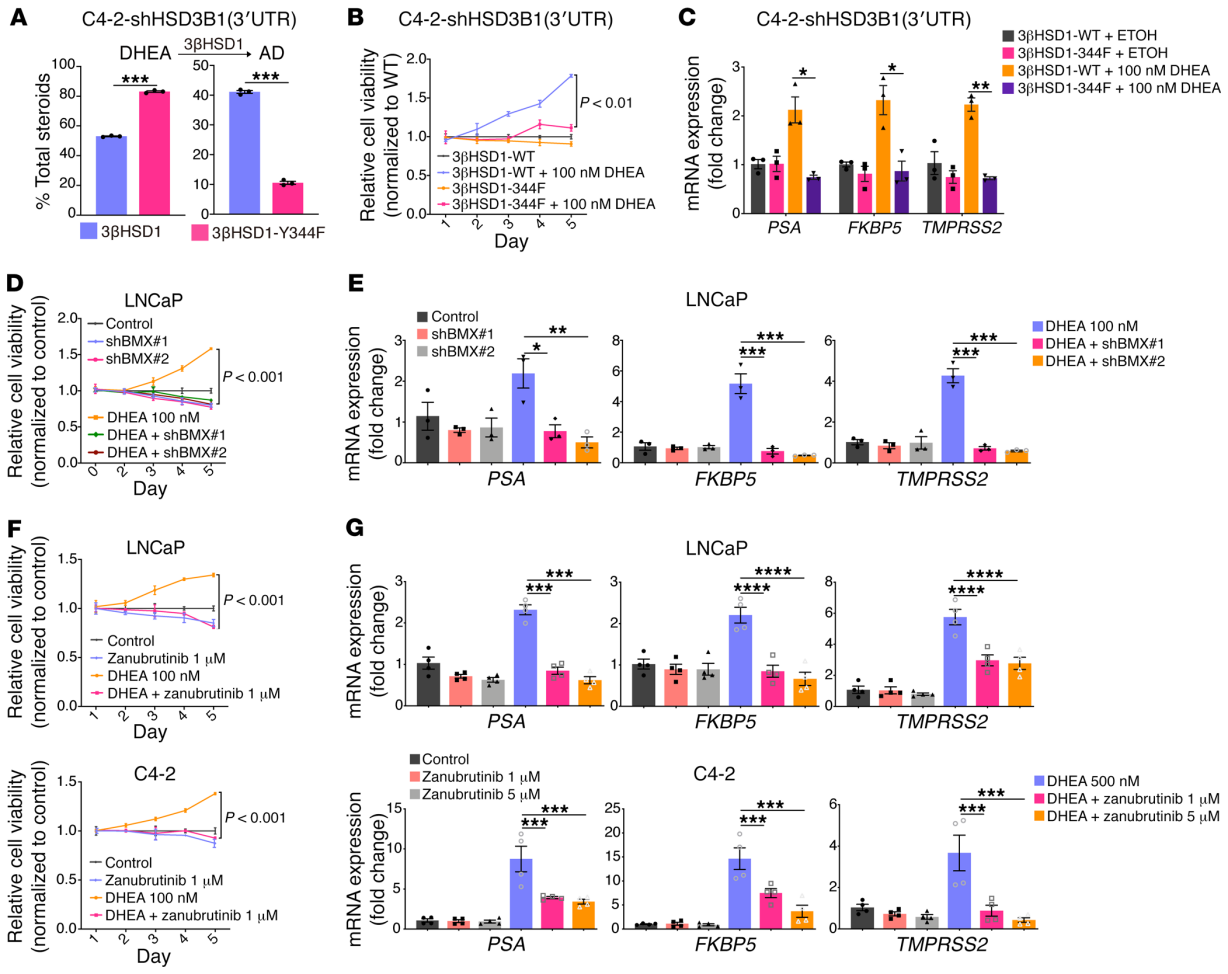
BMX blockade and inhibition of 3βHSD1 phosphorylation inhibit expression of androgen-regulated genes and prostate cancer proliferation. (**A**) C4-2 cells with stable shRNA-mediated knockdown of 3βHSD1 were stably infected with lentivirus expressing either WT 3βHSD1 or 3βHSD1-Y344F and subsequently treated with [^3^H]-DHEA for 5 hours, followed by steroid extraction from media and steroid separation and quantitation with HPLC. (**B**) As in **A**, but cells were deprived of serum overnight, followed by treatment with DHEA for the indicated days; cell proliferation was assessed with the WST-1 assay and growth for each cell line was normalized to WT control for each designated day. (**C**) As in **A**, but cells were deprived of serum overnight and treated with DHEA for 48 hours, followed by RNA extraction and qPCR. Expression is normalized to control and *RPLP0* expression. (**D**) Cells stably expressing shNT or 1 of 2 shRNA sequences against BMX were deprived of serum overnight, followed by treatment with DHEA and cell proliferation assessment as in **B**. (**E**) As in **C**, except that RNA extraction and qPCR was done on shBMX or control cells treated with DHEA for 48 hours. (**F**) LNCaP or C4-2 cells were deprived of serum overnight, treated with zanubrutinib or DHEA for the indicated times, and cell proliferation assessed as in **B**. (**G**) LNCaP or C4-2 cells were deprived of serum overnight and treated with zanubrutinib or DHEA for 48 hours, followed by RNA extraction and qPCR. Expression is normalized to control and *RPLP0* expression. For **A** and **C**, mean ± SEM represents combined data from 3 biological independent replicates performed in technical triplicate (unpaired 2-tailed *t* test). For **B**, **D**, and **F**, mean ± SEM represents 3 replicates in 1 experiment. Three independent experiments were performed (2-way ANOVA with Bonferroni’s multiple comparisons test). For **E**, mean ± SEM represents combined data from 3 biological independent replicates performed in technical triplicates (1-way ANOVA with Bonferroni’s multiple comparisons test). For **G**, mean ± SEM represents 4 replicates in 1 experiment. Three independent experiments were performed (1-way ANOVA with Bonferroni’s multiple comparisons test). *P < 0.05, **P < 0.01, ***P < 0.001, ****P < 0.0001.

We next investigated the biological function of 3βHSD1 pY344. To determine whether phosphorylation of 3βHSD1 affects prostate cancer cell growth, we produced C4-2 cell lines that stably expressed WT 3βHSD1 or 3βHSD1-Y344F. DHEA metabolism was retarded in the 3βHSD1-Y344F cell line (Figure 4A). We then tested cell viability and proliferation using the WST-1 assay and found that the Y344F mutation of 3βHSD1 inhibits DHEA-stimulated cell proliferation (Figure 4B). Further, the 3βHSD1 Y344F mutation inhibited AR-dependent transcriptional activity of canonical AR-regulated genes (Figure 4C). Our results suggest that 3βHSD1 Y344 phosphorylation promotes prostate cancer cell progression.

To explore whether targeting regulatory kinases can inhibit prostate cancer cell proliferation, we generated LNCaP cells stably expressing shNT or 2 shRNA sequences against BMX. After overnight serum deprivation, we assessed proliferation in the presence or absence of DHEA. The results indicated that knocking down BMX inhibited cell proliferation that is induced by DHEA (Figure 4D). In addition, qPCR results showed that knocking down BMX reduced DHEA-induced AR transcriptional activity (Figure 4E). Based on these findings, we hypothesized that BMX inhibitors also inhibit prostate cancer proliferative activity. We treated LNCaP and C4-2 cells with zanubrutinib and detected cell proliferation and AR target gene expression. Our results demonstrated that zanubrutinib effectively inhibited proliferation (Figure 4F) and AR transcriptional activity in the presence of DHEA (Figure 4G) in LNCaP and C4-2 cells. We also treated LNCaP and C-42 cells with another BMX inhibitor, ibrutinib, and assessed viability using Trypan blue staining (Supplemental Figure 4, A and B), the results of which were consistent with the cell proliferation results. To further address the specificity of BMX inhibitors we performed experiments with BMX overexpression in LNCaP and C4-2. The effects of BMX overexpression on 3βHSD1 phosphorylation, DHEA metabolism, AR signaling, and cell proliferation are all reversible with BMX pharmacologic inhibition (Supplemental Figure 4, C–F). In conclusion, our findings suggested that the phosphorylation of 3βHSD1 Y344 promoted prostate cancer proliferation, and targeting BMX as its regulatory kinase blocked the growth of prostate cancer cells.

### 3βHSD1-Y344F blocked CRPC growth in vivo.

**Figure 5:**
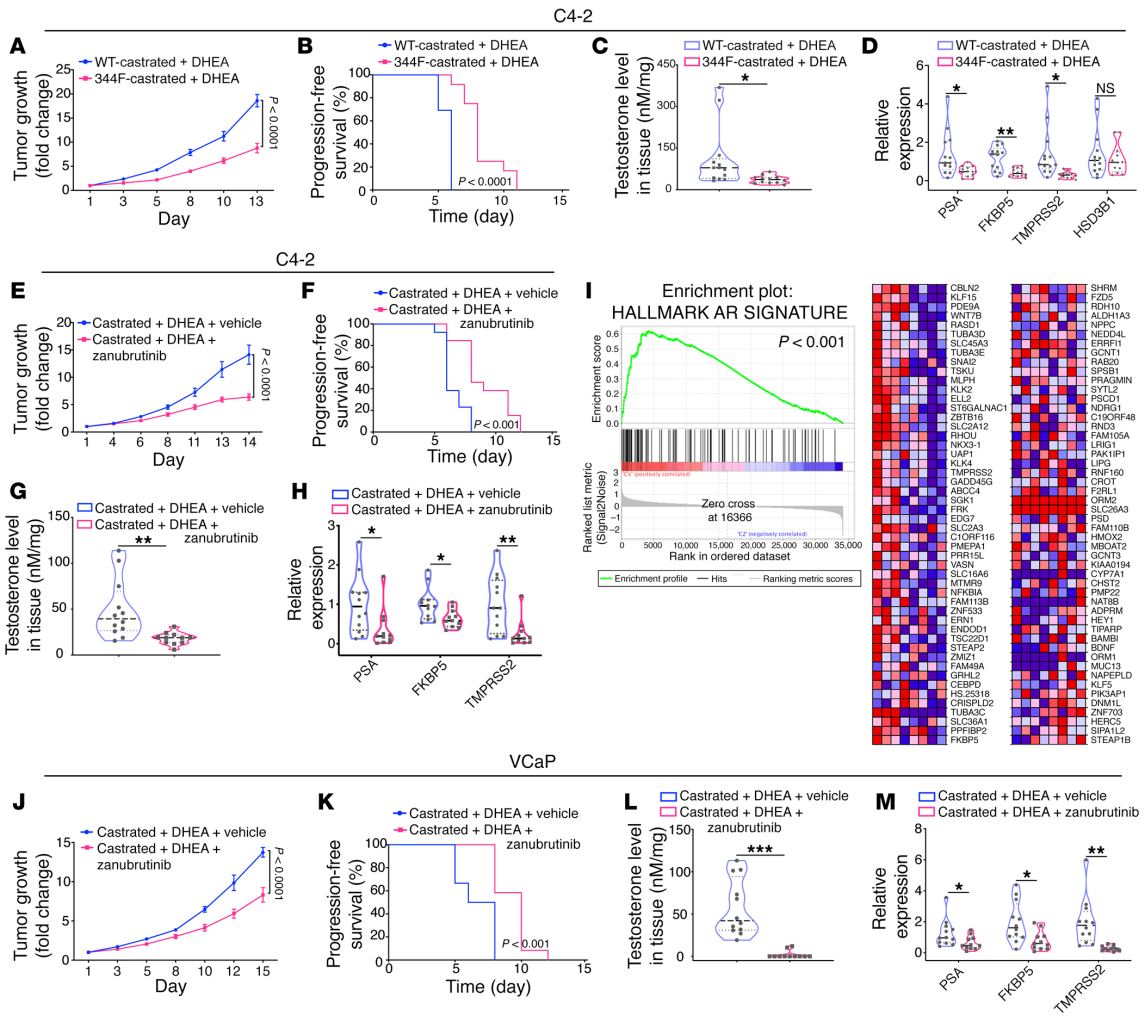
BMX pharmacologic blockade and 3βHSD1-Y344F inhibit androgen biosynthesis and CRPC growth in vivo. (**A**) C4-2 cells with stable shRNA-mediated knockdown of 3βHSD1 were stably infected with a lentivirus expressing WT 3βHSD1 or 3βHSD1-Y344F. Mice were subcutaneously injected with 10 million cells, and castration plus DHEA pellet implantation were performed after tumors reached 200 mm^3^. Tumor growth is shown as fold change from the time of treatment initiation for each tumor. The numbers of mice in the WT-3βHSD1/castration and 344F-3βHSD1/castration groups were 13 and 12, respectively. (**B**) Progression-free survival was assessed as time to 3-fold increase in tumor volume from treatment initiation. (**C**) The testosterone concentration in xenograft tumors was detected by mass spectrometry. (**D**) RNA was extracted from xenograft tumors, and expression of AR-responsive genes (*PSA*, *FKBP5*, and *TMPRSS2*) and *HSD3B1* was determined by qPCR. Expression was normalized to control and *RPLP0* expression. (**E**) 6 million C4-2 cells were injected subcutaneously in mice, and castration, DHEA-pellet implantation, and treatment with vehicle or zanubrutinib at a dose of 15 mg/kg by oral gavage twice daily was performed after tumors reached 150 mm^3^. Tumor growth was assessed as fold change from time of treatment initiation. The numbers of mice in the castration/vehicle and castration/zanubrutinib groups were 13 and 12, respectively. (**F**) Progression-free survival was assessed as time to 3-fold increase in tumor volume from treatment initiation. (**G**) Tumor testosterone in xenograft tumors was detected by mass spectrometry. (**H**) Expression of AR-regulated genes was assessed by qPCR and expression was normalized to control and *RPLP0* as in **D**. (**I**) RNA-Seq and GSEA was performed, showing AR inhibition as the top upstream regulator predicted to be inhibited. (**J**) Ten million VCaP cells were injected subcutaneously. Castration, DHEA-pellet implantation, and vehicle or zanubrutinib at a dose of 15 mg/kg by oral gavage twice daily were performed after tumors reached 200 mm^3^, and fold change in tumor volume from the time of treatment initiation was assessed. The numbers of mice in the castration/vehicle and castration/zanubrutinib groups were 12 and 11, respectively. (**K**) Progression-free survival was assessed as in **B**. (**L**) Xenograft testosterone detection by mass spectrometry. (**M**) AR-regulated genes were assessed as in **D**. For **A**, **E**, and **J**, *P* values were calculated by 2-way ANOVA with Bonferroni’s multiple comparisons test. For **B**, **F**, and **K**, *P* values were calculated with a log-rank test. For **C**, **D**, **G**, **H**, **L**, and **M**, *P* values were calculated using an unpaired 2-tailed *t* test. *P < 0.05, **P < 0.01, ***P < 0.001.

We next sought to determine the requirement for 3βHSD1 Y344 phosphorylation in development of CRPC in vivo in mouse xenograft models. We generated C4-2 cell lines that stably expressed WT 3βHSD1 or 3βHSD1-Y344F and subcutaneously injected the cells into NSG male mice to develop tumors, followed by surgical castration and subcutaneous implantation of 90-day sustained-release DHEA pellets to mimic the human physiology that occurs with ADT and CRPC development — similar to our prior studies (26–28). We found that the Y344F mutation of 3βHSD1 inhibited DHEA-induced tumor growth and prolonged progression-free survival in C4-2 xenograft models of CRPC (Figure 5, A and B). Mass spectrometry assessment of androgens in xenograft tissues (Figure 5C) demonstrated that the Y344F mutation inhibited tumor growth through loss of intratumoral androgen production. Reduction in AR transcriptional activity also was detected in xenograft tumors carrying mutated Y344F (Figure 5D). In contrast, the Y344F mutation had no significant effect on growth, tissue testosterone, or androgen signaling in xenografts growing in eugonadal mice (Supplemental Figure 5, A–D).

### Pharmacologic BMX blockade inhibited androgen biosynthesis and CRPC growth in vivo.

To investigate whether targeting phosphorylation blocked prostate cancer growth in vivo, we determined how effectively zanubrutinib blocked CRPC growth using mouse xenograft models. We established C4-2 or VCaP CRPC tumors with castration and DHEA pellet implantation. The mice were treated with zanubrutinib at a dose of 15 mg/kg or vehicle by oral gavage twice daily. Treatment with zanubrutinib led to significant tumor growth inhibition in both models compared with the vehicle control (*P* < 0.0001) (Figure 5, E and J). In contrast, zanubrutinib had virtually no effect on growth and androgen signaling in xenografts growing in eugonadal mice (Supplemental Figure 5, E and J). Differences in progression-free survival were similarly significant for zanubrutinib treatment in mice with CRPC (Figure 5, F and K; *P* < 0.0001) but not xenografts grown in the eugonadal mice (Supplemental Figure 5, F and K). Tumors from zanubrutinib-treated mice with CRPC displayed lower androgen production and AR target gene (*PSA*, *FKBP5*, *TMPRSS2*) expression (Figure 5, G, H, L, and M). By contrast, zanubrutinib had no significant effect on untreated tumors (Supplemental Figure 5, G and H and L and M). Unbiased RNA-Seq and Gene Set Enrichment Analysis (GSEA) similarly demonstrated that zanubrutinib inhibited AR-regulated genes (Figure 5I). Zanubrutinib had no significant effect on AR regulation in eugonadal tumors (Supplemental Figure 5E).

### Effects of BMX inhibition on 3βHSD phosphorylation and androgen synthesis in fresh human prostate tissues.

**Figure 6:**
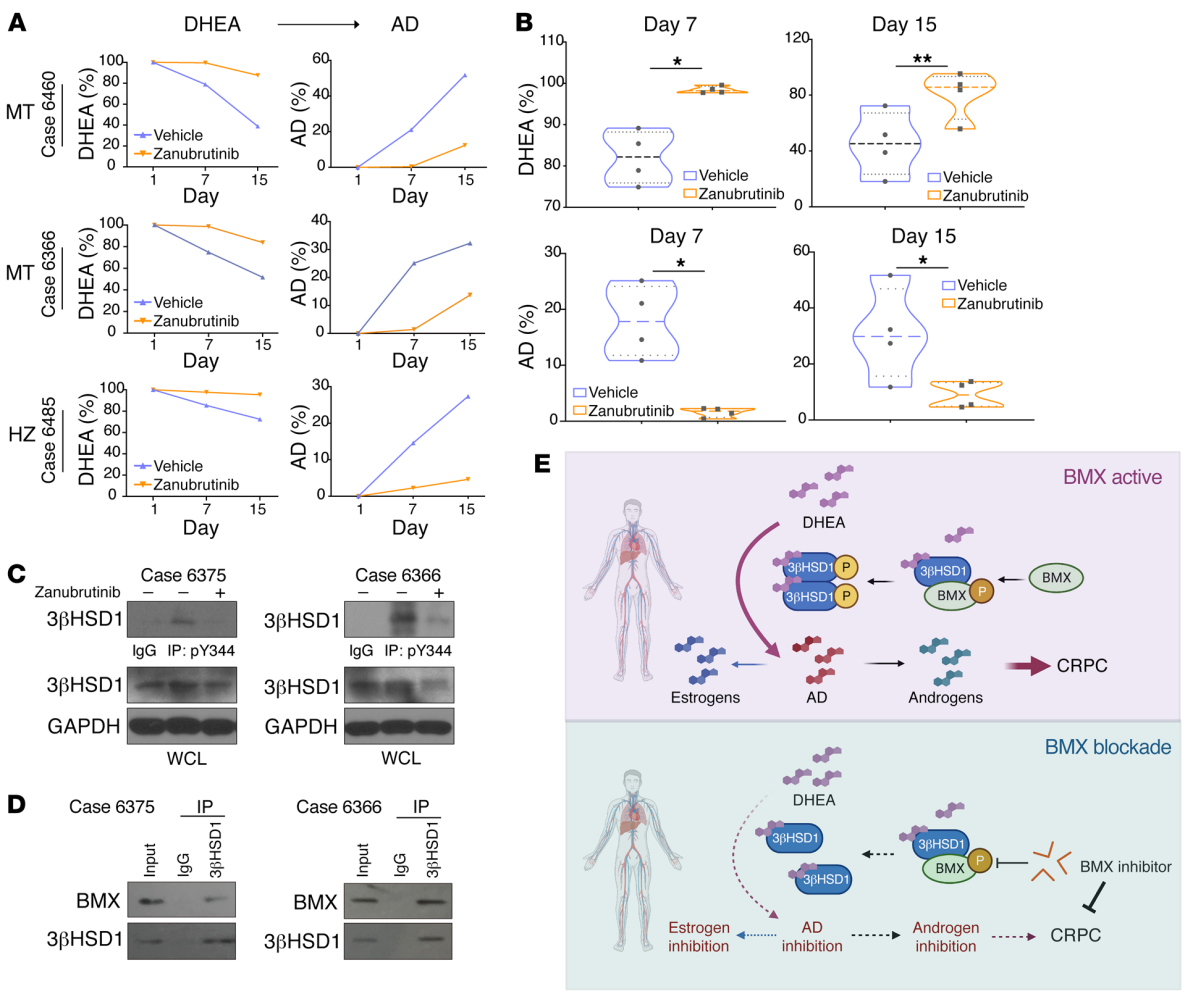
Targeting BMX inhibits phosphorylation and enzymatic activity of 3βHSD1 in prostate tissue of prostate cancer patients. (**A**) Fresh prostate tissues from 3 representative patients with prostate cancer from a total of 7 patients in whom DHEA metabolism was detectable; DHEA metabolism was analyzed in a total of 42 patients (MT, homozygous *HSD3B1*(1245C); HZ, heterozygous). Tissues were obtained and aliquoted in 2 equal portions. 1 portion was treated with zanubrutinib and the other with DMSO. Both portions were maintained in 3 ml DMEM containing 10% FBS, incubated for 12 hours, and then [^3^H]-DHEA was added to each portion. Cell culture medium was collected at the indicated times, and HPLC was performed. (**B**) DHEA metabolism was analyzed on day 7 and day 15. Mean ± SEM represents DHEA metabolism from 4 patients. *P < 0.05, **P < 0.01 (unpaired 2-tailed *t* test). (**C**) Protein was extracted from about 20 mg patient tissue, followed by 3βHSD1 immunoprecipitation and Western blot. (**D**) The remaining tissue was used for Western blot: tissue cores were minced and aliquoted in 2 equal parts and treated as in **A**. After 12 hours of culture, DHEA (10 nM) was added to each portion. 7 days later, protein was collected, and immunoprecipitation and Western blot were performed. (**E**) Proposed model for 3βHSD1 phosphorylation. BMX phosphorylates 3βHSD1 Y344 upon activation by DHEA. Y344 phosphorylation enhances 3βHSD1 activity by increasing its dimerization, which subsequently promotes androgen production and prostate cancer proliferation. When BMX is inhibited, 3βHSD1 phosphorylation–stimulated dimerization is lost, reducing cellular enzyme activity, potent androgen production, and prostate cancer proliferation. 3βHSD1 inhibition also blocks estrogen synthesis. For all panels, error bars represent the SEM; *P* values were calculated using paired 2-tailed *t* tests.

We next investigated the effects of BMX pharmacologic blockade in fresh prostate tissues cultured for metabolic assessment from men undergoing radical prostatectomy. Tissues were treated with [^3^H]-DHEA with or without zanubrutinib, and HPLC was performed on steroids extracted from medium to detect metabolism of steroids downstream of 3βHSD1. Tissues from a total of 7 patients, all of whom harbored the adrenal-permissive *HSD3B1* allele (3 homozygous and 4 heterozygous) had detectable DHEA metabolism and thus were assessable for reversibility with zanubrutinib. Notably, our results showed that the metabolism of DHEA was inhibited by zanubrutinib in all 7 prostate tissues (Figure 6, A and B and Supplemental Figure 6). Immunoprecipitation and Western blot results showed that both phosphorylation of 3βHSD1 and metabolic flux from DHEA to AD were reduced by inhibiting BMX with zanubrutinib (Figure 6C). In addition, the interaction between BMX and 3βHSD1 was observed in human prostate tissues (Figure 6D). Taken together, these results showed the potential therapeutic effects of targeting 3βHSD1 phosphorylation using BMX inhibitors for the treatment of men with CRPC (Figure 6E), and more generally, the role of BMX in physiologic regulation of extragonadal sex steroid biosynthesis.

## Discussion

Androgen dependence is a major hallmark of prostate cancer, even after progression on hormonal therapy (29, 30). Clinical responses to ADT are almost always followed by development of CRPC, due in large part to the fact that tumors engage in metabolic mechanisms to make their own potent androgens from extragonadal precursor steroids (1, 31). In the absence of gonadal testosterone, as is the case with ADT, the adrenals are the major source of precursors for sex steroids, and *HSD3B1* encodes the peripherally expressed enzyme that is necessary for conversion of DHEA to biologically active androgens and estrogens (32–34). Genetic evidence from at least 10 prostate cancer cohorts on inheritance of the adrenal-permissive *HSD3B1*(1245C) allele demonstrates that increased metabolic flux through 3βHSD1 hastens androgen biosynthesis, progression to CRPC, and prostate cancer mortality (12, 13, 35–38). Importantly, 3βHSD1 catalyzes steroid Δ^5^ → Δ^4^ isomerization and 3β-OH → 3-keto oxidation – reactions that are absolutely required for all pathways from the starting structure of (Δ^5^, 3β-OH) cholesterol or adrenal DHEA to testosterone and DHT (39). This may explain why treatment with abiraterone or enzalutamide does not overcome the adverse clinical outcomes conferred by the sustained androgen biosynthesis of adrenal-permissive *HSD3B1* inheritance as determined in studies from 4 institutions and over 800 men (15-17). Together, the existing clinical data suggest that direct inhibition of 3βHSD1 is necessary as a pharmacologic maneuver. However, until now, there has been no clinically appropriate mechanistic strategy to effectively pharmacologically block 3βHSD1 and to reverse the adverse clinical biology of the adrenal-permissive form of 3βHSD1 that is inherited by about half of all men with prostate cancer.

This study is the first to our knowledge to identify a posttranslational modification that is necessary for 3βHSD1 activity in cells. The BMX kinase is known to be upregulated with ADT (19). However, the mechanisms downstream of BMX, effects on tumor metabolism, and the context for contributions to CRPC have remained elusive. Our studies establish an essential role for BMX in regulating extragonadal sex steroid biosynthesis and suggest that the adverse clinical biology and poor survival in men with adrenal-permissive *HSD3B1* inheritance can be directly reversed by inhibiting BMX. Because they are in the same family of TEC nonreceptor kinases, there is a major overlap between tyrosine kinase inhibitors of BTK and BMX (20, 40). The specificity of BMX for interaction with 3βHSD1, in contrast to SRC family kinases, suggests the possibility of a requirement for the pleckstrin homology domain, which is present in BMX and other TEC family kinases (41, 42). Largely attributable to clinical advances in B cell leukemias for the purpose of BTK inhibition, several options for BTK/BMX inhibitors are available for clinical trials, including ibrutinib, zanubrutinib, alacabrutinib and abivertinib (43, 44). Based on our mechanistic findings here, a multi-institutional phase 2 clinical trial of abivertinib plus abiraterone is underway for men with metastatic CRPC who inherit the adrenal-permissive *HSD3B1* allele (NCT05361915). This trial is poised to test an entirely new mechanistic concept for the treatment of metastatic CRPC in a disease and treatment space where, to this point, the use of kinase inhibitors have not been shown to be effective (45). Unlike other solid tumors — such as melanoma and lung cancer, where kinase inhibitors have been successful — activating kinase mutations that are bona fide genetic drivers are generally uncommon in human prostate cancer. Instead, in this instance, kinase activity is required for the adrenal-permissive 3βHSD1 enzyme, which is directly and mechanistically linked to treatment resistance and prostate cancer lethality in multiple human cohorts.

Finally, 3βHSD1 also lies 1 step upstream of aromatase, which is required for the generation of estrogens. The Δ^4^, 3-keto-steroid products of 3βHSD1, AD and testosterone, are converted to estrone and estradiol, respectively (24, 46). Similar to the context of men who are absent gonadal testosterone, emerging evidence suggests an essential role for *HSD3B1* inheritance in postmenopausal women who have only adrenal precursors as their physiologic source of sex steroids. The frequency of the homozygous adrenal-permissive *HSD3B1* genotype is enriched in women with estrogen-driven postmenopausal breast cancer and occurs in about 15% of these tumors (47). Furthermore, these women with homozygous adrenal-permissive *HSD3B1* inheritance have a significantly increased rate of metastatic recurrence after treatment for localized breast cancer, even with hormonal therapy (48), suggesting that these women have more aggressive disease and that new strategies are required to improve clinical outcomes. Our data suggest that 3βHSD1 inhibition and AD suppression impedes estrogen biosynthesis in the postmenopausal setting and that BMX inhibition is a strategy that should be tested in clinical trials.

In conclusion, BMX is required for phosphorylation of 3βHSD1 and the synthesis of potent androgens from extragonadal precursor steroids. These mechanistic data suggest a clinical pharmacologic strategy to counter the aggressive disease and poor clinical outcomes conferred by inheritance of the adrenal-permissive *HSD3B1* allele, which commonly occurs in men with prostate cancer. Further, BMX inhibition may play a role in blocking estrogen biosynthesis in women with estrogen-driven postmenopausal breast cancer. Our findings have broad applications to treatment of sex-steroid-dependent diseases and understanding essential mechanisms of normal physiology and its variations.

## Methods

### Antibodies, chemicals, and reagents

#### Antibodies.

Mouse monoclonal antibodies against 3βHSD1 (1:2,000, ab55268) and rabbit polyclonal antibodies against phospho-BMX (1:2,000, ab59409) were purchased from Abcam. Mouse monoclonal antibodies against phospho-tyrosine (pTyr) (1:2,000, 05-1050) were purchased from Sigma-Aldrich. custom-made rabbit monoclonal antibodies against phospho-3βHSD1 Y344 (1:2,000) were ordered from Affinity Biosciences. Mouse monoclonal antibodies against GST (1:5,000, AE001) were purchased from Abclonal. Mouse monoclonal antibodies against Flag (1:5,000, F3165) and anti-Flag M2 affinity gel (A2220) were purchased from Sigma-Aldrich. Rabbit monoclonal antibodies against HA (1:3,000, 3724S), β-actin (1:3,000, 3700S), and rabbit polyclonal antibodies against BMX (1:3,000, 24773) and GAPDH (1:5,000, 14C10) were obtained from Cell Signaling Technology.

#### Chemicals.

Zanubrutinib (BGB-3111), ibrutinib (S2680), and acalabrutinib (ACP-196) were purchased from Selleckchem. The kinase inhibitor library was obtained from the Lerner Research Institute Molecular Screening Core. [^3^H]-labeled DHEA (100 nM, 300,000–600,000 cpm) was purchased from PerkinElmer, and steroids were purchased from Steraloids.

#### Reagents.

Puromycin (A1113803) and hygromycin (10687010) were bought from Thermo Fisher Scientific. DNA transfection reagent FuGENE HD (E2311) was purchased from Promega. GelCode Blue Stain Reagent (24590) was obtained from Pierce.

### Cell lines and constructs

LNCaP and C4-2 cells were purchased from ATCC and cultured in RPMI 1640 medium with 10% FBS (Gemini). VCaP, JEG3, and 293T cells were purchased from ATCC and cultured in DMEM containing 10% FBS. LAPC4 cells were a gift from Charles Sawyers (Memorial Sloan Kettering Cancer Center, New York, New York) and were maintained in Iscove’s modified Dulbecco’s medium with 10% FBS and 1% penicillin-streptomycin (Gibco).

Constructs of shRNAs targeting BMX (5′-GCAATATGACAGCAACTCAAA-3′; 5′-GATCACAATCTGAACAGTTAC-3′) and *HSD3B1* (5′-GAAGGTTTCTGTCCTAATCAT-3′) were purchased from Sigma-Aldrich. These constructs were used to generate the BMX knockdown LNCaP stable cell lines or *HSD3B1* knockdown C4-2 stable cell lines by using a lentiviral system. 293T cells (ATCC) were cotransfected for 48 hours with 10 μg each of the constructed plasmid, pMD2.G, and psPAX2 vector to package the virus. The virus was then concentrated by using PEG-it Virus Precipitation Solution (System Biosciences) according to the provided protocol. Next, LNCaP or C4-2 cells were infected with the concentrated virus for 24 hours with addition of polybrene (10 g/ml), followed by selection with puromycin (2 μg/ml) for approximately 2 weeks.

pCMV5-HA-HSD3B1 was provided by J. Ian Mason (retired, University of Edinburgh, Edinburgh, United Kingdom) (49), sequenced, and confirmed as encoding for 3βHSD1(367T). PCR-amplified 3βHSD1(367T) was cloned into pCMV-Flag and pCDH-GST. The plasmid encoding mutated 3βHSD1(Y344F, Y255F, Y340F) was derived using the Quick Change Site Directed Mutagenesis kit (Agilent Technologies). For C4-2 cells stably expressing WT 3βHSD1 or 3βHSD1-Y344F, the WT or mutant was PCR-amplified and sub-cloned into the pLVX-Flag-Puro vector. Lentiviral particles were packaged in 293T cells by cotransfecting 10 μg each of pLVX-Flag-Puro vector, pMD2.G, and psPAX2 vector. Next, C4-2 cells with stable shRNA-mediated knockdown of 3βHSD1 were stably infected with the concentrated virus for 24 hours with addition of polybrene (10 μg/ml), followed by selection with hygromycin (2 μg/ml) for approximately 2 weeks.

A guide RNA sequence for targeting *HSD3B1* 5′-CGTTTATACTAGCAGAAAGGC-3′ was designed and cloned, and virus was produced using the LentiCRISPRv2 protocol (50).

### Steroid metabolism

Cells (300,000–400,000 cells per well) were seeded and maintained in 12-well plates that were coated with poly L-ornithine (Sigma-Aldrich) for 12 hours and then treated with [^3^H]-DHEA (100 nM, 300,000–600,000 cpm; PerkinElmer). Cells were incubated at 37°C and aliquots of medium (0.3 ml) were collected at the indicated time points. Collected media was incubated with 300 U β-glucuronidase (*Helix pomatia*; Novoprotein) at 37°C for at least 2 hours, extracted with 600 μL 1:1 ethyl acetate/isooctane, and concentrated under a nitrogen stream.

For HPLC analysis, the concentrated samples were dissolved in 50% methanol and injected on a Breeze 1525 system equipped with a model 717 plus autoinjector (Waters Corp). Steroid metabolites were separated on a Luna 150 × 4.6 mm, 3 μm C18 reverse-phase column (Phenomenex) using methanol/water gradients at 30°C. The column effluent was analyzed using a β-RAM model 3 in-line radioactivity detector (IN/US Systems Inc.) with Liquiscint scintillation mixture (National Diagnostics). All metabolism studies were performed in triplicate and repeated in independent experiments.

### Gene expression

Total RNA was extracted with GenElute Mammalian Total RNA miniprep kit (Sigma-Aldrich), and 1 μg RNA was reverse-transcribed to cDNA with the iScript cDNA Synthesis Kit (Bio-Rad). An ABI 7500 real-time PCR instrument (Applied Biosystems) was used to perform quantitative PCR (qPCR) analysis, using iTaq Fast SYBR Green Supermix with ROX (Bio-Rad) in 96-well plates at a final reaction volume of 10 μL. The qPCR analysis was carried out in triplicate with the following primer sets: *HSD3B1*, 5′-CCATGTGGTTTGCTGTTACCAA-3′ (forward), 5′-TCAAAACGACCCTCAAGTTAAAAGA-3′ (reverse); *PSA,* 5′-GCATGGGATGGGGATGAAGTAAG-3′ (forward), 5′-CATCAAATCTGAGGGTTGTCTGGA-3′ (reverse); *FKBP5,* 5′-CCCCCTGGTGAACCATAATACA-3′ (forward), 5′-AAAAGGCCACCTAGCTTTTTGC-3′ (reverse); *TMPRSS2,* 5′-CCATTTGCAGGATCTGTCTG-3′ (forward), 5′-GGATGTGTCTTGGGGAGCAA-3′ (reverse); *RPLP0* (large ribosomal protein P0, a housekeeping gene), 5′-CGAGGGCACCTGGAAAAC-3′ (forward, 5′-CACATTCCCCCGGATATGA-3′ (reverse).

For steroid-treated cells, each mRNA transcript was quantitated by normalizing the sample values to *RPLP0* and to vehicle-treated cells. All gene expression studies were repeated in at least 3 independent experiments.

### Immunoblots and immunoprecipitation

For immunoblots, total cellular protein was extracted with ice-cold RIPA lysis buffer (Sigma-Aldrich) containing protease inhibitors (Roche) and phosphatase inhibitors (Sigma-Aldrich). Protein concentration was determined using a BCA protein assay (Pierce Protein Research Products, Thermo Fisher Scientific). Protein, 30–50 μg, was separated by 10% SDS-polyacrylamide gel electrophoresis and then transferred to a nitrocellulose membrane (Millipore). After incubating overnight at 4°C with the anti-pTyr antibody, anti-BMX antibody, anti-p-BMX antibody, anti-3βHSD1 antibody, anti-GST antibody, or anti-flag antibody as appropriate, the appropriate secondary antibody was added and incubated for 1 hour at room temperature. A chemiluminescent detection system (Thermo Fisher Scientific) was used to detect the bands with peroxidase activity. An anti-GAPDH antibody (1:5,000; G9545, Sigma-Aldrich) was used as a control for sample loading. An additional preIP control lysate was used for experiments involving an IgG control.

For immunoprecipitation of HA- or GST-tagged 3βHSD1, cell lysates (2 mg) were incubated with 30 μL anti-HA or anti-GST affinity gel overnight at 4°C. Beads were washed with lysis buffer 4 times and samples were then used for immunoblotting with phospho-Tyr or phospho-3βHSD1-Y344. Protein lysates, 50 μg each, were loaded on an SDS-polyacrylamide gel. For coimmunoprecipitation of 3βHSD1 and kinase, 293T cells (at 60% confluence) were transfected with 5 μg GST-tagged 3βHSD1 and HA-tagged kinase for 36 hours. Immunoprecipitation was performed as described above. See complete unedited blots in the supplemental material.

### Mass spectrometry analysis of 3βHSD1 phosphorylation

Using an anti-GST affinity gel, GST-tagged 3βHSD1 was immunoprecipitated from C4-2 cells treated with DHEA (10 nM) for 1 hour. The precipitated complexes were boiled at 95°C for 10 minutes. GST-tagged 3βHSD1 was separated from the complexes by SDS-PAGE and then trypsinized. The GST-tagged 3βHSD1 band was excised from the gel as closely as possible and washed and destained in 50% ethanol, 5% acetic acid. The gel pieces were then dehydrated in acetonitrile, dried in a Speed-vac, and digested by adding 5 μL trypsin (10 ng/μL) in 50 mM ammonium bicarbonate, followed by incubation overnight. The peptides were extracted into 2 portions of 30 μL each 50% acetonitrile, 5% formic acid. The combined extracts were evaporated to <10 μL in a Speed-vac and then resuspended in 1% acetic acid to make up a final volume of approximately 30 μL for liquid chromatography–mass spectrometry (LC-MS/MS) analysis.

The LC-MS/MS system was a Thermo Fisher Scientific Orbitrap Elite system. The HPLC column was a Dionex 15 cm × 75 μm id Acclaim PepMap C18, 2 μm, 100 Å reversed-phase capillary chromatography column. 5 microliters of the extract volume was injected, and the peptides, eluted from the column in an acetonitrile, 0.1% formic acid gradient at a flow rate of 0.25 μL/min, were introduced into the source of the mass spectrometer online. The micro-electrospray ion source was operated at 2.5 kV. The digest was analyzed in both a survey manner and a targeted manner. The survey experiments were performed using the data-dependent multitask capability of the instrument, acquiring full scan mass spectra to determine peptide molecular weights and product-ion spectra to determine amino acid sequences in successive instrument scans. The LC-MS/MS data were searched with the program Sequest (bundled into Proteome Discoverer 2.3) against both the human UniProtKB database (downloaded on February 28, 2019; 20,429 entries) and specifically against the sequence of GST-tagged 3βHSD1. The parameters used in this search include a peptide mass accuracy of 10 ppm, fragment ion mass accuracy of 0.6 Da, carbamidomethylated cysteines as a constant modification, and oxidized methionine and phosphorylation at S, T, and Y as a dynamic modification. The results were filtered to a peptide and protein level FDR rate of less than 1% using a target decoy strategy. All positively identified phosphopeptides were manually validated. The targeted experiments involved the analysis of specific GST-tagged 3βHSD1 peptides. The chromatograms for these peptides were plotted based on known fragmentation patterns, and the peak areas of these chromatograms were used to determine the extent of phosphorylation (51, 52).

### In vitro kinase assay

In brief, GST-3βHSD1 and HA-BMX were purified from 293T cells. 3βHSD1 was dephosphorylated by incubating with alkaline phosphatase at 37°C, then incubated with or without BMX in kinase buffer (60 mM HEPES pH 7.5, 5 mM MgCl_2_, 5 mM MnCl_2_, 3 μM Na_3_VO_4_ and 1.25 mM DTT). ATP, 20 μM, was added to the kinase buffer to start the reaction. The reactions were performed in a total volume of 50 μL at 30°C for 30 minutes and then terminated by adding SDS-PAGE loading buffer.

### Enzyme kinetics

293T cells were transfected with Flag-3βHSD1 or Y344F mutant with or without cooverexpressed HA-BMX. AFter 48 hours, 3βHSD1 or 3βHSD1-Y344F mutant was purified using the FLAG M Purification Kit (Sigma-Aldrich) according to the protocol provided by the manufacturer. Briefly, cell pellets were washed with 10 volumes of PBS and centrifuged. Cells were suspended in CelLytic M reagent and incubated for 20 minutes on ice. The cells were then centrifuged, and the supernatant was loaded onto the prepared column, which included anti-FLAG M2 affinity gel under gravity flow. The column was then washed with 10 column volumes of 1 × wash buffer to remove unbound proteins, and then 3βHSD1 protein was eluted with 1 ml of 1 × wash buffer containing 3 × FLAG peptide (200 ng/ml). The Flag peptides were removed, and proteins were concentrated using an Amicon Ultra-0.5 centrifugal filter concentrator (Millipore) and quantitated by BCA protein assay (Pierce). 1 μg Flag-3βHSD1 or Y344F protein was subjected to SDS-PAGE. The protein purity was verified by GelCode Coomassie blue stain reagent (Pierce) following the instructions of the manufacturer and also verified by Western blotting. For Coomassie blue staining, the gel was washed with deionized water for 15 minutes and then incubated with GelCode stain reagent for 1 hour followed by ultrapure water for 1 hour.

To detect the kinetics of 3βHSD1, an NAD+ turnover assay was performed. Preparations containing DHEA (1–20 μM), NAD+ (0.1 mM) and 1 μg protein in 0.25 ml of 50 mM potassium phosphate (pH 7.4) were incubated for 1 hour before using the Promega NADH detection kit. After incubating an additional hour, luminescence was measured using a BioTek Synergy Neo Multi-Mod Plate Reader (BioTek). The Km and Kcat were calculated by Michaelis-Menten analysis with nonlinear regression using GraphPad Prism software.

### Cell proliferation assay and cell counting

Cells (approximately 10^4^/well) were plated in triplicate in 96-well plates coated with poly dl-ornithine, incubated overnight, then starved with phenol red–free medium containing 5% dextran-treated charcoal stripped FBS for 48 hours and treated with 100 nM DHEA and combined with indicated drug treatments for 5 days, then assayed using the Cell Proliferation Reagent WST-1 (Sigma-Aldrich). Absorbance was normalized to controls as indicated.

Cells (approximately 10^5^/well) were plated in triplicate in 12-well plates coated with poly dl-ornithine, incubated overnight, then starved with phenol red-free medium containing % dextran-treated charcoal stripped FBS for 48 hours and treated with 100 nM DHEA and combined with indicated drug treatments for 5 days; viable cells were assessed using Trypan blue (Thermo Fisher Scientific) and counted using a cell counter (Thermo Fisher Scientific).

### Mouse xenograft studies

All NOD SCID γ (NSG) male mice (6–8 weeks old) were purchased from the Jackson Laboratory. Between 6 and 10 million cells were injected subcutaneously in mice. After tumors reached 150–200 mm^3^, mice were surgically orchiectomized and implanted with 5 mg 90-day sustained-release DHEA pellets to mimic human adrenal DHEA production in men with CRPC.

To evaluate WT 3βHSD1 or 3βHSD1-Y344F C4-2 cell growth in vivo, 10 million WT 3βHSD1 or 3βHSD1-Y344F stable C4-2 cells (100 μL in 50% Matrigel and 50% growth media) were subcutaneously injected into mice. When tumors reached 200 mm^3^ (length × width × width × 0.52), the mice were arbitrarily placed into 2 groups: eugonadal or castration plus DHEA treatment. Tumor volume was measured every other day, and progression-free survival was assessed as time to 3-fold increase in tumor volume from the time tumors reached 200 mm^3^. The numbers of mice in the WT 3βHSD1/eugonadal, WT 3βHSD1/castration, 3βHSD1-Y344F/eugonadal or 3βHSD1-Y344F/castration groups were 12, 13, 11, and 12, respectively. The number of mice in each group was determined by those that survived surgical procedures and had reached the 200 mm^3^ tumor volume required to initiate treatment. Tumor diameters were measured by digital calipers 3 times per week. Tumors were fresh frozen upon mouse sacrifice.

To evaluate whether zanubrutinib suppressed tumor growth, 6 million C4-2 or 10 million VCaP cells (100 μL in 50% Matrigel and 50% growth media) were subcutaneously injected into mice. When tumors reached 150 (C4-2) or 200 (VCaP) mm^3^ (length × width × width × 0.52), the mice were arbitrarily divided among 4 groups: eugonadal/vehicle (safflower seed oil [Sigma-Aldrich] with 10% DMSO), eugonadal/zanubrutinib (15 mg/kg in safflower seed oil with 10% DMSO), castration/vehicle, and castration/zanubrutinib. The mice were given vehicle or zanubrutinib by oral gavage twice daily. Tumor volume and progression-free survival were determined as described above. The numbers of mice in the C4-2 eugonadal/vehicle, eugonadal/zanubrutinib, castration/vehicle, and castration/zanubrutinib groups were 11, 12, 13, and 12, respectively. The numbers of mice in the VCaP eugonadal/vehicle, eugonadal/zanubrutinib, castration/vehicle, and castration/zanubrutinib groups were 11, 11, 12, and 11, respectively. The number of mice in each group was determined by those that survived surgical procedures and had reached the 200 mm^3^ tumor volume required to initiate treatment. Tumor diameters were measured by digital calipers 3 times per week and fresh frozen upon mouse sacrifice.

### Mass spectrometry analysis

#### Xenograft tissues.

Androgens in xenografts were assessed by LC-MS/MS as reported previously with slight modifications (26, 27). In brief, at least 30 mg tumor tissue was homogenized with 500 μL liquid chromatography-mass spectrometry–grade water (Thermo Fisher Scientific) using a homogenizer. The mixture was then centrifuged at 15,000*g* for 10 minutes at 4°C. Supernatant was transferred to a glass tube, followed by the addition of 25 μL internal standard (d_3_-T). The steroids and the internal standard were extracted with 2 mL methyl tert butyl ether (Across) evaporated to dryness under nitrogen and then reconstituted with 200 μL 50% methanol.

### Estrogens and androgens in cell culture

#### Steroid extraction.

Freshly collected media samples were frozen and kept at –80°C until the LC-MS/MS analysis. For the analysis, a 250 μL media sample was spiked with 10 μL internal standards mix [5 ng/mL of E2-^13^C_3_, 25 ng/mL, androstene-3, 17-dione-2,3,4-^13^C_3_ and 5α-dihydrotestosterone-d3 (16,17,17-d3)] in a glass tube. The steroids were extracted using methyl-tert-butyl ether (MTBE, Across) using liquid-liquid extraction. The combined MTBE fractions were dried under a gentle nitrogen gas flow. Then the dried sample was reconstituted with 120 μL of 50% methanol (methanol/water [v/v]). The reconstituted sample was divided in 2 fractions, 1 for estrogen and 1 for androgen analyses.

#### Estrogen analysis.

An ultra-high pressure liquid chromatography (NEXERA X2, Shimadzu Corporation) system with a C18 column (InfinityLab Poroshell 120 EC-C18 column, 4.6 × 75 mm, 2.7 μm, Agilent) and gradient was used to separate estrogens in 1 of the prepared fractions. The separated estrogens were selected and quantified by mass spectrometry (Qtrap 5500, AB Sciex) by using multiple reaction monitoring (MRM) mode in negative ion electrospray ionization (ESI).

#### Androgen analysis.

The other prepared fraction was injected onto the UPLC system, and the androgens were separated on a C18 column (Zorbax Eclipse Plus C_18_ column, 150 mm × 2.1 mm, 3.5 μm, Agilent). A gradient was used. The separated androgens were quantified on the Qtrap 5500 mass spectrometer using the MRM mode in positive ion ESI. MultiQuant Software (version 3.0.3, AB Sciex) was used for the data acquisition and quantification for estrogens and androgens.

### RNA-Seq analysis

Tumor RNA was extracted from the mice used in the C4-2 zanubrutinib treatment experiment, 4 samples from each group. RNA was extracted with GenElute Mammalian Total RNA miniprep kit (Sigma-Aldrich). The Case Western Reserve University Genomics Core performed the RNA-Seq using the HumanHT-12 v4 Expression BeadChip and iScan (Illumina). Hybrid signals were analyzed with Illumina GenomeStudio Software 2011.1 and normalized to the vehicle control group. Heatmaps were generated with HemI software (version 1.0). GSEA was used to correlate the 5α-Abi expression data with an androgen receptor-selective gene set described elsewhere (53). The GSEA enrichment plot was generated as described elsewhere (54).

### Human tissue studies

Fresh prostate tissue cores (60–100 mg) from 42 patients were obtained for germline DNA analysis and DHEA metabolism.

#### DHEA metabolism studies.

Between 40 and 60 mg of tissue was used for DHEA metabolism detection. Briefly, tissue cores were minced and aliquoted into 2 equal portions. One was treated with zanubrutinib, and the other was treated with DMSO. Both tissues were maintained in 3 ml DMEM containing 10% FBS and incubated in a 5% CO_2_ humidified incubator. After 12 hours of culture, [^3^H]-DHEA was added to each portion. Cell culture medium was collected at the indicated time points, and HPLC was performed as described above. Protein was extracted from approximately 20 mg of human prostate tissue, followed by 3βHSD1 immunoprecipitation and Western blot. To determine the effects of BMX inhibition, the remaining 20–40 mg tissue was minced and aliquoted into 2 equal parts. 1 portion was treated with zanubrutinib, and the other was treated with DMSO. Both tissues were maintained in 3 ml DMEM containing 10% FBS and incubated in a 5% CO_2_ humidified incubator. After 12 hours of culture, 10 nM DHEA was added to each portion. After 7 days, tissue was homogenized with ice-cold RIPA lysis buffer (Sigma-Aldrich) containing protease inhibitors (Roche) and phosphatase inhibitors (Sigma-Aldrich) using a homogenizer to extract protein; immunoprecipitation and Western blot were then performed.

#### Genotyping studies.

A total of 42 clinical prostate tissues were obtained. Germline DNA was genotyped for *HSD3B1* as described previously (14), and 19, 18, and 5 cases had 0, 1, and 2 copies of the adrenal-permissive *HSD3B1*(1245C) allele, respectively. Of these, 0 of 19, 4 of 18, and 3 of 5 were observed to have DHEA metabolism, and the 7 showing metabolism were included to assess the effects of zanubritinib on 3βHSD1 metabolic activity.

### Data and materials availability

All data needed to evaluate the conclusions in the paper are available in the main text or the supplemental materials.

### Statistics

Statistical data analyses were performed in GraphPad Prism software (version 9.0.0) and Microsoft Excel (version 16.43). In general, for mouse xenograft studies, progression-free survival was determined by Kaplan-Meier analysis followed by a log-rank test to compare among groups. For other comparative analyses, an unpaired 2-tailed *t* test was used unless otherwise noted. *P* < 0.05 was considered statistically significant.

### Study approval

All mouse studies were performed under a protocol approved by the IACUC of the Cleveland Clinic Lerner Research Institute. All human tissues were obtained at the Cleveland Clinic under IRB-approved protocols. All human tissues were deidentified, and all patients provided written informed consent.

## Author contributions

NS and XL were responsible for conceptualization of the study. NS, XL, MB, CG, MA, YC, CW, and BW were responsible for developing the methodology and data acquisition. NS and XL were responsible for the data analysis. XL, MB, CG, MA, YC, CW, MP, NR, ZZ, and BW were responsible for experimentation. NS, SS, and EK provided resources for the project. NS and XL were responsible for writing the original draft, and all authors participated in reviewing and editing subsequent drafts and final submission. Funding was provided by NS, XL, and MB All authors have read and approved the manuscript.

## Supplementary Material

Supplemental data

## Version 1 01/17/2023

Electronic publication
