# Supplementary material for: BMX controls 3βHSD1 and sex steroid biosynthesis in cancer
Source: J Clin Invest. 2023 Jan 17;133(2):e163498. doi: 10.1172/JCI163498 (PMC9843047; doi:10.1172/JCI163498)
Supplement: Supplemental data [file jci-133-163498-s007.pdf]

Supplementary Data

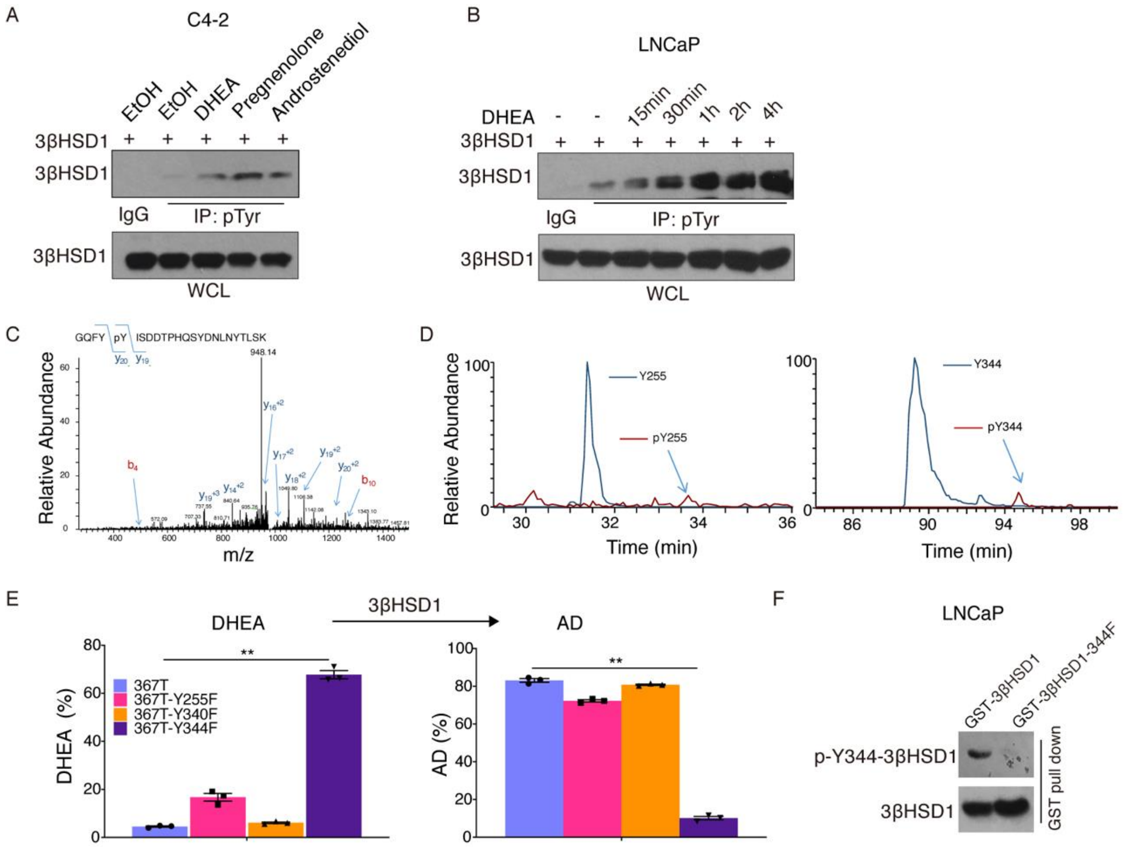

Supplemental Fig. 1. 3βHSD1 phosphorylation. A. C4-2 cells overexpressing HA-3βHSD1 were treated with the indicated steroids (10 nM) for 1 hour. Pan-phospho-tyrosine (pTyr) was detected by immunoprecipitation and western blot. **B.** LNCaP cells overexpressing HA-3βHSD1 were treated with DHEA (10 nM) for the indicated times. Pan-phospho-tyrosine (pTyr) was detected by immunoprecipitation and western blot. Lysate from cells expressing HA-3βHSD1 and no DHEA treatment were used for the IgG control. **C.** 3βHSD1-GST was transfected in C4-2 cells, and cells were treated with DHEA for 1 hour. GST pull-down complexes were immunoblotted, and the indicated phosphopeptides on human 3βHSD1 were identified by LC-MS/MS. A triply charged peptide with a mass of 983.75 Da was identified in a targeted analysis of GST-HSD3B1. The CID spectra for this peptide are dominated by doubly charged C-terminal y ions. The mass difference between y_{19} and y_{20} is consistent with modification at Y255. **D.** Chromatograms for the unmodified, Y255, and pY255 peptides from GST-HSD3B1 are shown as are chromatograms for the unmodified, Y344, and pY344 peptides from GST-HSD3B1. **E.** HA-3βHSD1 enzyme activity was assessed by determining DHEA metabolism with HPLC. Cells were transfected with HA-3βHSD1 mutants and subsequently treated with $[^3H]$-DHEA for 6 hours, followed by steroid extraction from media and steroid separation and quantitation with HPLC. Mean ± SEM represents

3 replicates in 1 experiment. Three independent experiments were performed. **P < 0.01 (1-way ANOVA test with Dunnett's multiple comparisons test). **F.** Validation of phospho-3βHSD1-Y344 antibody.

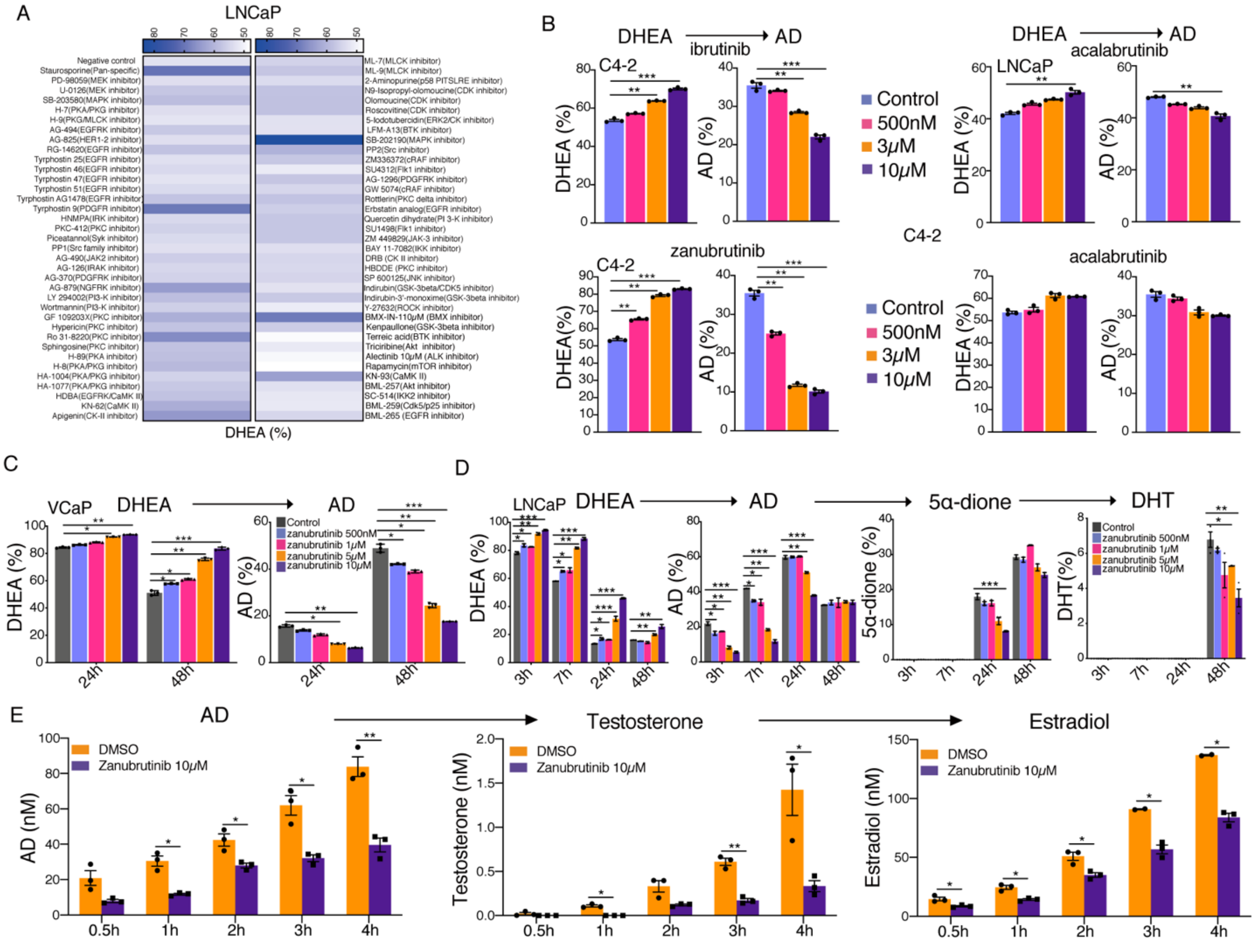

Supplemental Fig. 2. Tyrosine kinase inhibition and regulation of cellular 3βHSD1 activity. A. BMX inhibition suppresses DHEA metabolism. Kinase screening was performed by detecting DHEA metabolism using a kinase inhibitor library. LNCaP cells were treated with about 88 kinase inhibitors (10 μM) for 1 hour and subsequently treated with [^{3}H]-DHEA for 5 hours. Medium was then collected, and steroids were extracted and separated and quantified with HPLC. Color scale indicates % DHEA remaining. **B.** LNCaP or C4-2 cells were treated with BMX inhibitors for 1 hour and subsequently treated with [^{3}H]-DHEA for 5 hours. Medium was then collected and steroids were extracted from medium and separated and quantified with HPLC. **C, D.** As in (B), but experiments were done in both LNCaP and VCaP cell lines using various concentrations of zanubrutinib for the indicated times to determine effects on DHEA metabolism. **E.** JEG3 cells were starved with RPMI-1640 medium containing 10% charcoal-stripped fetal bovine serum; 24 hours later, 10 μM zanubrutinib was added, followed by DHEA 16 hours later. After DHEA addition, cells were cultured for the indicated times, medium was collected, and mass spectrometry was performed to detect the steroid level. For B, mean ± SEM represents 3 replicates in 1 experiment. Three independent experiments were performed (1-way ANOVA test with Dunnett's

multiple comparisons test). For C and D, mean ± SEM represents combined data from 3 biological independent replicates performed in technical triplicate (1-way ANOVA test with Dunnett's multiple comparisons test). For E, mean ± SEM represents 3 replicates in 1 experiment. Three independent experiments were performed (multiple unpaired t-tests). * $P < 0.05$, **$P < 0.01$, *** $P < 0.001$, **** $P < 0.0001$.

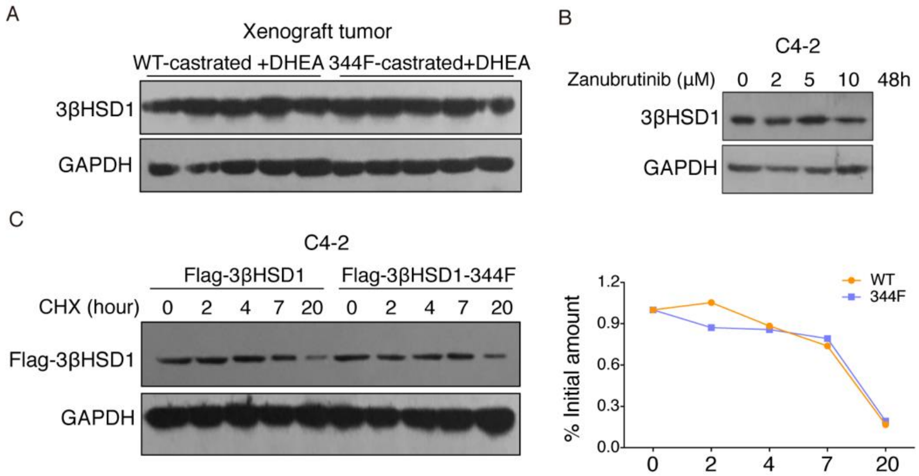

Supplemental Fig. 3. Effects of 3βHSD1-Y344F mutation on protein levels. A. C4-2 cells with stable shRNA-mediated knockdown of 3βHSD1 were stably infected with a lentivirus expressing 3βHSD1 (WT) or 3βHSD1-Y344F grown in castrated mice after tumors reached 200 mm^3. Proteins were extracted from tumor tissue, and western blot was performed. **B.** C4-2 cells were treated with 2, 5, or 10 μM zanubrutinib for 48 hours. Proteins were extracted, and western blot was performed. **C.** Flag-tagged WT or Y344F-3βHSD1 was transfected into C4-2 cells; then cells were treated with cyclohexamide (CHX) for the indicated times, followed by protein extraction, western blot, and protein quantitation.

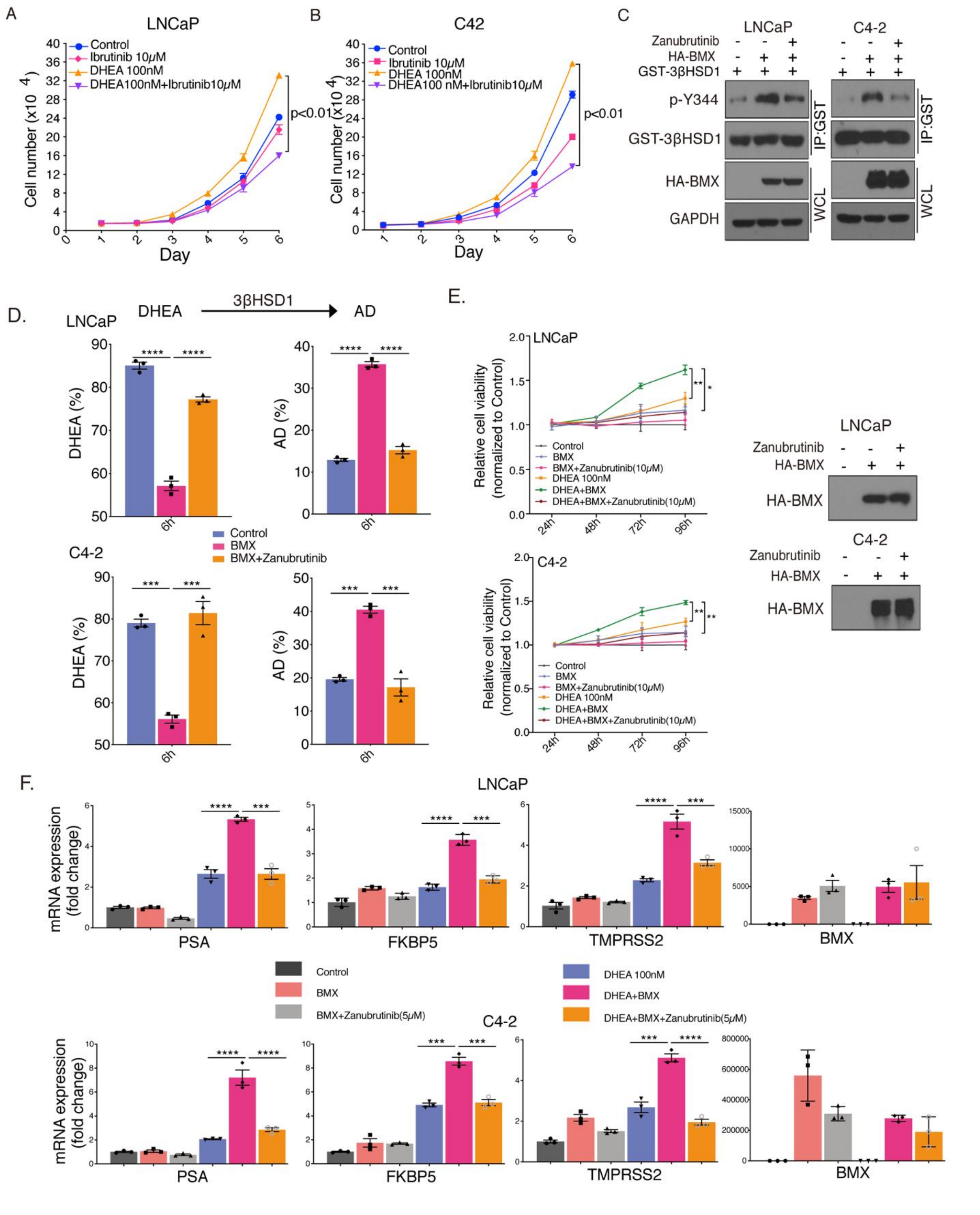

Supplemental Fig. 4. Effects of BMX inhibition on cell viability. A. LNCaP cells were deprived of serum overnight, followed by treatment with ibrutinib or DHEA for the indicated times, and the number of viable cells was determined by Trypan blue exclusion assay. **B.** C4-2 cells were

deprived of serum overnight, followed by treatment with ibrutinib or DHEA for the indicated times, and the viable cell number was determined with Trypan blue. **C.** Cells overexpressing 3βHSD1 or BMX were treated with zanubrutinib for 3 hours, and DHEA for 1 or 2 hours, respectively. Phospho-3βHSD1-Y344 was detected by immunoprecipitation and western blot. **D.** Cells overexpressing BMX were treated with zanubrutinib for 3 hours and subsequently treated with [^{3}H]-DHEA for 6 hours, followed by steroid extraction from media and steroid separation and quantitation with HPLC. **E.** As in (D), but cells were deprived of serum overnight, followed by treatment with DHEA for the indicated days; cell proliferation was assessed with the WST-1 assay, and growth for each cell line was normalized to WT control for each designated day. Proteins were lysed for 96 hours, then cells were collected and western blot was performed to detect the effect of BMX overexpression. **F.** As in (D), but cells were deprived of serum overnight and treated with DHEA for 48 hours, followed by RNA extraction and qPCR. Expression is normalized to control and *RPLP0* expression. For A and B, mean ± SEM represents combined data from 3 biological replicates performed in technical triplicate (2-way ANOVA test with Bonferroni's multiple comparisons test). For D and F, mean ± SEM represents 3 replicates in 1 experiment. Three independent experiments were performed (1-way ANOVA test with Bonferroni's multiple comparisons test). For E, mean ± SEM represents combined data from 3 biological replicates performed in technical triplicate (2-way ANOVA test with Tukey's multiple comparisons test).

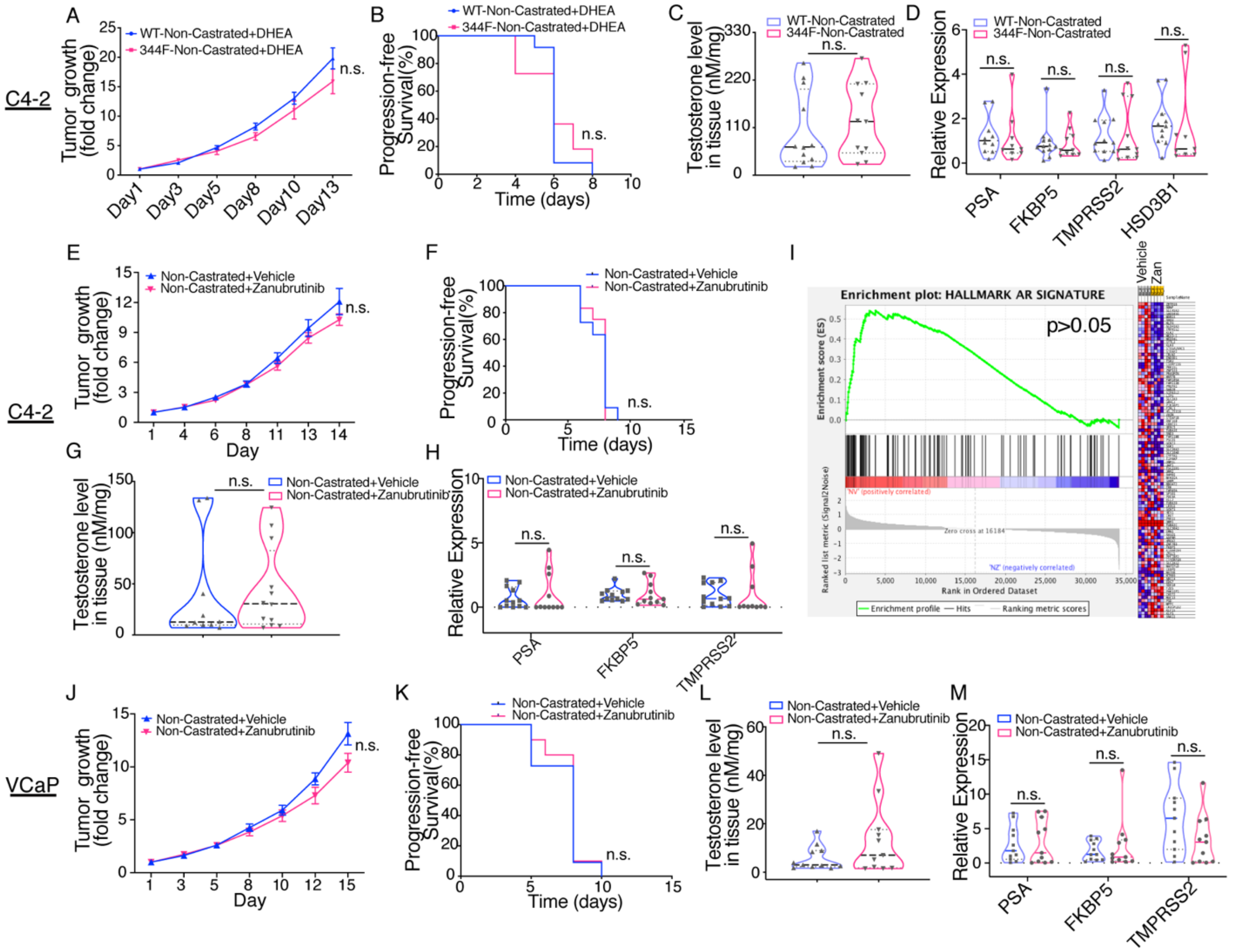

Supplemental Fig. 5. The effect of pharmacologic BMX inhibition and 3βHSD1-Y344F on xenografts in eugonadal mice. A. C4-2 cells with stable shRNA-mediated knockdown of 3βHSD1 were stably infected with a lentivirus expressing 3βHSD1 (WT) or 3βHSD1-Y344F. Mice were subcutaneously injected with 10 million cells. Tumor growth is shown as fold change from the time when tumors reached 200 mm^3. The numbers of mice in the WT-3βHSD1/eugondal and 344F-3βHSD1/eugonadal groups were 12 and 11, respectively. **B.** Progression-free survival was assessed as time to 3-fold increase in tumor volume from the time tumors reached 200 mm^3. **C.** Testosterone concentration in xenograft tumors was detected by mass spectrometry. **D.** RNA was extracted from xenograft tumors and gene expression was determined by qPCR. **E.** Six million C4-2 cells were injected subcutaneously in mice. After tumors reached 150 mm^3, zanubrutinib or vehicle treatment was started. Tumor growth was analyzed. The numbers of mice in the eugonadal/vehicle and eugonadal/zanubrutinib groups were 11 and 12 respectively. **F.** progression-free survival was assessed as time to 3-fold increase in tumor volume from treatment initiation. **G.** Absolute concentration of testosterone in xenograft tumors was detected by mass spectrometry. **H.** RNA was extracted from xenograft tumors, and AR target gene expression was determined by qPCR. Expression is normalized to control and *RPLP0* expression. **I.** RNA-seq was performed

with 4 tumors from each group and Gene Set Enrichment Analysis was done for the AR signature gene set. **J.** Ten million VCaP cells were injected subcutaneously in mice, and treatment with vehicle or zanubrutinib was initiated when tumor volume reached 200 mm^3. There were 11 mice in each cohort for the eugonadal/vehicle and eugonadal/zanubrutinib groups. **K.** Progression-free survival was assessed as time to 3-fold increase in tumor volume from treatment initiation, and the significance of the difference between groups was calculated with a log-rank test. **L.** Testosterone in xenograft tumors was detected by mass spectrometry. **M.** AR target gene expression was determined as in (H). For A,E, and J, *P* values were calculated with 2-way ANOVA test with Bonferroni's multiple comparisons test. For B.F.K. *P* values were calculated with a log-rank test. For C, D, G, H, L, and M, *P* values were calculated using an unpaired 2-tailed t-test. n.s, no significant difference.

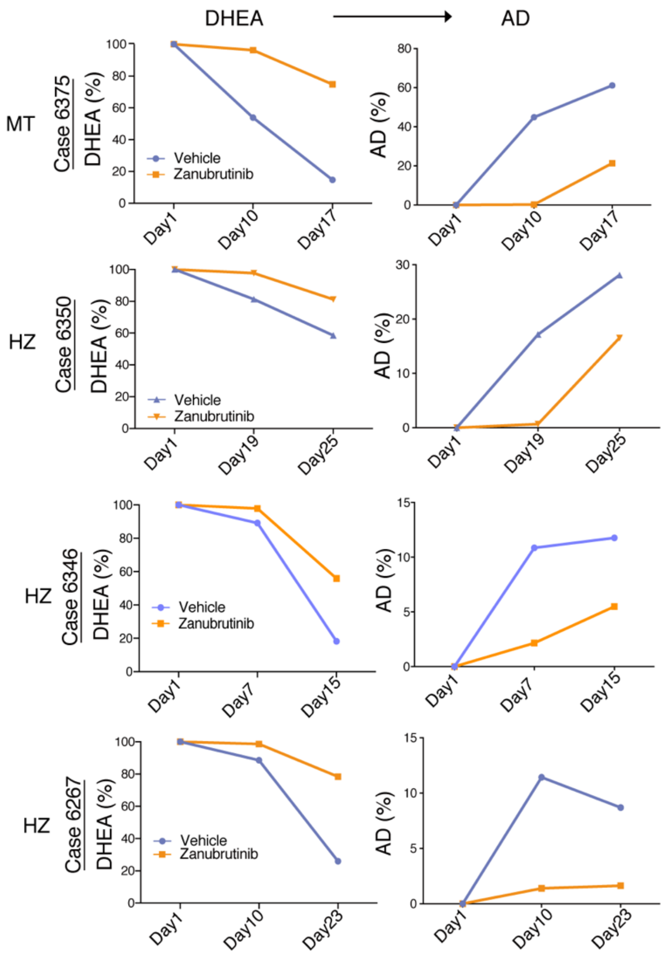

Supplemental Fig. 6. Effects of zanubrutinib on 3βHSD1 activity in human prostate tissue. Fresh prostate tissue cores (40 - 60 mg) from 4 patients were obtained, minced, and aliquoted in two equal portions. One was treated with zanubrutinib, and the other was treated with DMSO. Both tissues were maintained in 3 mL DMEM containing 10% fetal bovine serum and incubated in a 5% CO_2 humidified incubator. After 12 hours of culture, [^{3}H]-DHEA was added to each portion. Cell culture medium was collected, and HPLC was performed.
